# Supplementary material for: Dynamic ambulance relocation: a scoping review
Source: BMJ Open. 2023 Dec 14;13(12):e073394. doi: 10.1136/bmjopen-2023-073394 (PMC10729233; doi:10.1136/bmjopen-2023-073394)
Supplement: Supplementary data [file bmjopen-2023-073394supp001.pdf]

Supplementary material 1; Search strategy

Search strategy for each database

Pubmed (NLM). Search performed October 12, 2023, 338 papers.

Ambulance, emergency medical services, dispatch, dynamic, dynamic allocation, relocation, machine learning		
	1.	Search: ((((dispatch*[Title/Abstract]) OR ("Emergency Medical Dispatcher"[Mesh])) OR ("Resource Allocation"[Mesh]) OR ("Emergency Medical Dispatch"[Mesh]) OR (allocation[Title/Abstract])) OR (relocation[Title/Abstract])) OR (reallocation[Title/Abstract]) OR (redeployment[Title/Abstract]) OR (deployment[Title/Abstract]) AND ((((((((((algorithm*[Title/Abstract]) OR (artificial intelligence[Title/Abstract])) OR (AI[Title/Abstract])) OR (machine learning[Title/Abstract])) OR (data-driven[Title/Abstract])) OR (data-based[Title/Abstract])) OR ("dynamic allocation"[Title/Abstract]) OR ("dynamic*"[Title/Abstract])) OR ("Algorithms"[Mesh])) OR ("Artificial Intelligence"[Mesh])) OR ("Machine Learning"[Mesh]))) AND ((((((ambulance*[Title/Abstract]) OR ("emergency medical service*"[Title/Abstract])) OR ("emergency service*"[Title/Abstract])) OR (prehospital[Title/Abstract])) OR ("Ambulances"[Mesh])) OR ("Emergency Medical Services"[Mesh]))
	2.	1 AND Filters: English, from 2012 - 2023

Web of Science Core Collection (Clarivate) (1975-present) Search performed October 19, 2023, 301 papers.

Indexes=SCI-EXPANDED, SSCI, A&HCI, CPCI-S, CPCI-SSH, ESCI Timespan=All years

Ambulance, emergency medical service		
	1.	ALL=(((ambulance OR emergency medical service)))
Dispatch		
	2.	ALL=((dispatch*OR allocation* OR reallocation* OR redeployment* OR deployment*))
Dynamic, relocation, machine learning		
	3.	ALL=(((dynamic* OR dynamic allocation OR relocation OR machine learning OR data-driven OR artificial intelligence OR Machine learning)))
Combined Sets		
	4.	1 AND 2 AND 3
Limits		
	6.	4 and English (Languages) and 2023 or 2022 or 2021 or 2020 or 2019 or 2018 or 2017 or 2016 or 2015 or 2014 or 2013 or 2012 (Publication Years)

Scopus (Elsevier). Search performed October 19, 132 abstracts

Ambulance, emergency medical services		
	1.	(TITLE-ABS-KEY (ambulance*) OR TITLE-ABS-KEY (emergency AND medical AND service*))
Dispatch		
	2.	TITLE-ABS-KEY (dispatch*)
Dynamic, relocation, machine learning		
	3.	(TITLE-ABS-KEY (dynamic*) OR TITLE-ABS-KEY (relocation*) OR TITLE-ABS-KEY (machine AND learning)) OR TITLE-ABS-KEY (dynamic AND allocation) OR TITLE-ABS-KEY (relocation) OR TITLE-ABS-KEY (machine learning) OR TITLE-ABS-KEY (data-driven) OR TITLE-ABS-KEY (artificial intelligence)))
Combined Sets		
	4.	1 AND 2 AND 3
Limits		
	8.	4 AND (LIMIT-TO (LANGUAGE , "English")AND PUBYEAR > 2011 AND PUBYEAR < 2024)

Google Scholar Search performed September 12 and October 20, 2023. Manual search.

Dynamic ambulance		
	1.	‘dynamic ambulance’
Ambulance relocation		
	2.	‘ambulance relocation’
	3.	‘dynamic ambulance dispatch’
Limits		
	4.	(Publication Years 2012-2023). Sorted: Best match.
