# Supplementary material for: Dynamic ambulance relocation: a scoping review
Source: BMJ Open. 2023 Dec 14;13(12):e073394. doi: 10.1136/bmjopen-2023-073394 (PMC10729233; doi:10.1136/bmjopen-2023-073394)
Supplement: Supplementary data [file bmjopen-2023-073394supp002.pdf]

Supplementary material 2: Quality appraisal according to CASP protocols

**Author/ year**	**Did the study address a clearly focused issue?**	**Was the cohort requited in an acceptable way?**	**Was the exposure accurately measured to minimize bias?**	**Was the outcome accurately measured to minimize bias?**	**Have the authors identified all important confounding factors?**	**Have con-founding factors in the design and/or analysis been taken into consideration?**	**Was the follow-up of subjects complete and long enough?**	**How precise are the results?**	**Do you believe in the results?**	**Can the results be applied to the local population?**	**Do the results of this study fit with other available evidence?**	**Overall quality**
Barneveldt 2017	Yes	N/A	N/A	Some	Some	Some	Not able to judge	Some	Some	Yes	Yes	Medium
Bélanger et al 2016	Yes	N/A	N/A	Yes	Some	Yes	Not able to judge	Some	Yes	Yes	Yes	Medium
Billhart et al 2014	Yes	N/A	N/A	Some	No	Some	Yes	Some	Yes	Yes	Yes	Medium
Carvallo et al 2020	Yes	N/A	N/A	Some	Some	Yes	Yes	Some	Yes	Yes	Yes	High
Degel et al 2015	Yes	N/A	N/A	Some	Some	Yes	Yes	Some	Yes	Yes	Yes	Medium
Deng et al 2021	Yes	N/A	N/A	Some	Some	Some	N/A	Some	Some	Unclear	Yes	Medium
Enayati et al 2018	Yes	N/A	N/A	Some	Some	Some	Yes	Some	Some	Yes	Yes	Medium
Hajiali et al 2022	Yes	N/A	N/A	Yes	Some	Yes	No	Good	Some	Yes	Some	High
Jagtenberg et al 2017	Yes	N/A	N/A	Some	Some	Yes	N/A	Some	Some	Unclear	Yes	Medium
Jánošíková et al 2021	Yes	N/A	N/A	Some	Some	Some	Not able to judge	Some	Some	Yes	Yes	Medium
Karpova et al 2022	Yes	N/A	N/A	Some	Some	Some	Not able to judge	Some	Some	Yes	Yes	Medium
Lam et al 2014	Yes	N/A	N/A	Yes	Yes	Some	Yes	Good	Yes	Yes	Yes	High
Lam et al 2015	Yes	N/A	N/A	Yes	Some	Some	Yes	Some	Yes	Yes	Yes	Medium
Lam et al 2017	Yes	N/A	N/A	Yes	Yes	Yes	Yes	Some	Yes	Yes	Yes	High
Nogueira et al 2016	Yes	N/A	N/A	Some	Some	Some	Some	Some	Some	Yes	Yes	Medium
Roa et al 2020	Yes	N/A	N/A	Some	Some	Some	Some	Some	Some	Yes	Yes	Medium
Schmid 2021	Yes	N/A	N/A	Some	Some	Some	Yes	Some	Some	Yes	Yes	Medium
Strauss et al 2021	Yes	N/A	N/A	Some	Some	Some	Unclear	Some	Yes	Yes	Yes	Medium

Swalehe & Aktas 2016	Yes	N/A	N/A	Some	Some	Some	Unclear	Some	Yes	Yes	Yes	Medium
Umam et al 2022	Yes	N/A	N/A	Some	Some	Some	Unclear	Some	Yes	Yes	Yes	Medium
Van Buuren et al 2018	Yes	N/A	N/A	Yes	Some	Yes	Yes	Good	Yes	Yes	Yes	High
