# Supplementary material for: Dynamic ambulance relocation: a scoping review
Source: BMJ Open. 2023 Dec 14;13(12):e073394. doi: 10.1136/bmjopen-2023-073394 (PMC10729233; doi:10.1136/bmjopen-2023-073394)
Supplement: Supplementary data [file bmjopen-2023-073394supp003.pdf]

Supplementary material 3. Overview of included studies

**Authors and publication year**	**Study location**	**Design**	**Aim of the study**	**Setting**	**Type of modelling**	**Variables used in the model**	**Outcome variable**	**Main findings**	**Quality appraisal**
Barneveldt et al 2017[1]	The Netherlands	Experimental	To develop a method to obtain good relocation strategies in real time	Rural areas	Mathematic models and scenario probabilities based on retrospective data	Demand locations<br>Time of the day<br>Level of priority<br>Total number of ambulances<br>Treatment time on scene	Response time	A one-step look-ahead heuristic model that relocated ambulances to minimize the response time was presented	Medium quality
Bélanger et al 2016[2]	Canada	Experimental with simulation	To study outcomes of different location and relocation strategies.	Urban areas	Mathematic models and scenario probabilities	Available ambulances<br>Travel times<br>Ambulance stations locations<br>Population density<br>Expected emergency calls<br>Working shift time	Response time<br>Response rate within 9 minutes<br>Travelled distance	Using a flexible relocation strategy provides a better service. However, when the system is congested, dynamic strategies cannot compensate for the lack of resources	Medium quality
Billhardt et al 2014[3]	Spain	Experimental	To present an event-driven system for dynamic ambulance assignment and redeployment	Urban areas	Mathematic models and scenario probabilities	Geographical location<br>Available ambulances<br>(The study only included critical patients)	Response time	Mathematic models can reduce the response times for EMS services. However, more variables need to be considered and included in such models	Medium quality

Carvalho et al 2020[4]	Portugal	Experimental with simulation	To develop models to solve ambulance dispatching and relocation problems	Urban areas	Mathematic models and scenario probabilities based on retrospective data (n=20 000)	Available ambulances<br>Travel times<br>Ambulance stations locations<br>Dynamic relocation spots<br>Waiting time<br>Maximum response times<br>Uncovered emergencies	Availability for new calls	A mixed-integrated programming model and an experimental model was presented. Both approaches used a time preparedness measure to evaluate the system's capability to handle new emergencies	High quality
Degel et al 2015[5]	Germany	Experimental with simulation	To support decision on location planning for ambulances	Rural areas	Mathematic models and scenario probabilities based on retrospective data	Travel time<br>Required coverage<br>Variation in fleet<br>Flexible locations	Availability for new calls	The inclusion of time-dependent and empirical data had a substantial impact on the solution	Medium quality
Deng et al 2021[6]	China	Experimental	To optimize the EMS system	No information given	Mathematic models	Population density<br>Road network<br>Location of hospitals<br>Location of flexible locations	Response time	The solution presented could effectively achieve a population coverage within 15 minutes, with a minimum number of temporary ambulance points in addition to existing	Medium quality

								ambulance stations	
Enayati et al 2018[7]	USA	Experimental with simulation	To develop a real-time model to maximize coverage with minimum possible total travel time	Urban	Mathematic models and scenario probabilities based on retrospective data (n=50 000)	Initial fleet size of ambulances<br>Travel limit (max 20 min)<br>Maximum allowed workload of each ambulance<br>Day and time<br>Shift times (12h)	Turnaround time	The modeling of the real time deployment model resulted in improvement in average coverage and prevented accumulated workload for ambulance personnel	Medium quality
Hajiali et al, 2022[8]	Iran	Experimental real-life testing	To develop a decision support system for optimal ambulance coverage	Urban	Mathematic models and real-life evaluation (1 week) of response time and workload for each ambulance	Base location<br>Demand zones and time ranges from these (7 min, 15 min, 25 min)<br>Number of available ambulances<br>Relocation time<br>Working shift time (12h)	Response time<br>Workload	The decision support system decreased response times and reduced the total working time of all ambulances by about 9% per shift.	Hight quality
Jagtenberg et al 2017[9]	The Netherlands	Experimental with simulation	To present different dispatch rules and suggest a heuristic method for ambulance dispatching	Urban areas	Mathematic models and scenario probabilities	Ambulance station locations<br>Demand locations<br>Hospital locations<br>the base location<br>Time of the day<br>Driving time	Response time	Two methods to obtain ambulance dispatch policies were reported. Their effects could not be defined	Medium quality
Jánošíková et al 2021[10]	Slovakia	Experimental with simulation	To identify optimization	Urban and rural areas	Mathematic models and	Demand zones<br>Population (and age profile)	Response time	The EMS systems should be designed to	Medium quality

			criterion of the EMS		scenario probabilities	Temporary locations Travel time		minimize response times, and not to maximize the number of calls served within a given time limit	
Karpova et al 2022 (2023)[11]	Spain	Experimental	To addresses the problem of dynamic relocation of ambulances through the design and development of heuristic tools	Urban and rural areas	Mathematic models and scenario probabilities	Population coverage in demand zones Ordinary base location Driving time Possible relocation bases	Response time	The designed relocation algorithms perform better than if there was no relocation strategy.	Medium quality
Lam et al 2014[12]	Singapore	Experimental with simulation	To develop a discrete-event simulation model for the Singapore EMS	Urban area	Mathematic models and scenario probabilities based on retrospective data (n=54 548)	Geographical information systems (GIS) data, Travel time Ambulance location	Response time	Response times were improved via a more effective reallocation of ambulances and dispatch policy, without additional ambulances	High quality
Lam et al 2015[13]	Singapore	Experimental with simulation	To develop efficient ambulance allocation plans in Singapore and to evaluate its	Urban area	Mathematic models and scenario probabilities based on retrospective data (n= 52 512)	Call times Dispatch times, Scene arrival times, Hospital conveyance times Turnaround time, Locations of incident	Response time	Geographical information system-based plans reduced the median response times with 13 to 44 seconds	Medium

			performance using the DES model.					compared to a static reallocation policy	
Lam et al 2017[14]	Singapore	Experimental with simulation	To report on the previously reported work by leveraging on the DES model	Urban area	Mathematic models and scenario probabilities based on retrospective data (n=217 000)	Geographical coverage<br>Response Times<br>Turnaround times and utilization rates	Turnaround time	The integration of dynamic redeployments optimized the ambulance coverage	High quality
Nogueira et al 2016[15]	Brazil	Experimental with simulation	To analyze the EMS using two modeling techniques: optimization and simulation	Urban areas	Mathematic models and scenario probabilities	Ambulance station location<br>Demand posts<br>Hospital locations<br>Cost for each ambulance	Response time	A balanced number of ambulances allocated across certain bases at certain times improved the system performance without increasing the total number of ambulances	Medium quality
Roa et al 2020[16]	Colombia	Experimental with simulation	To design an algorithm to support the real-time operation of ambulances	Urban areas	Mathematic models and scenario probabilities based on retrospective data	Types of emergency,<br>Types of ambulances<br>Demand points<br>Locations for ambulances<br>Modelled on-site attention time<br>Travel times	Response time<br>Availability for new calls	The real-time management of ambulance fleets involved challenging decisions due to frequent, complex, and random changes in system conditions	Medium quality

Schmid 2021[17]	Austria	Experimental with simulation	To propose a stochastic dynamic model for the ambulance relocation and dispatching problem	Urban area	Mathematic models and scenario probabilities based on retrospective data (n=3748)	Location of hospitals and patients, response times, travel times, using data.	Response time	By deviating from the traditional rule of dispatching the closest ambulance available and relocating them to their home base after having finished serving, the coverage can be improved	Medium quality
Strauss et al 2021[18]	Switzerland and Germany	Experimental with simulation	To explain the major features of “rule-based discrete event simulation”	Urban and rural areas	Mathematic models and scenario probabilities	Response times, demands per day, travel time, dispatch priority	Response time	Rule-based DES models can improve regional emergency services’ efficiency without increasing cost. All simulation-based methods suggest normative solutions and optimize EMS’ performance within given healthcare system structures	Medium quality
Swalehe & Aktas 2016[19]	Turkey	Experimental	To find optimal ambulance	Urban	Computer based systems (one with	Time of day Population Driving time	Response time	Ambulance response time can be reduced	Medium quality

			locations (stations and temporary demand points)		information on retrospective emergency calls	Road network data		with a dynamic relocation plan taking time of the day in consideration. Dynamic ambulance deployment is a more effective ambulance deployment strategy than static ambulance deployment	
Umam et al 2022[20]	Indonesia	Experimental	To overcome the location problem and allocation of ambulances by using a combination of metaheuristics and simulation	No information given	Mathematic models	Ambulance station location<br>Demand posts<br>Travel times<br>Number of available ambulances	Response time	Involving several potential bases can produce a short response time.	Medium quality
Van Buuren et al 2018[21]	The Netherlands	Experimental real-life testing	To evaluate two dynamic relocation policies for operational use and implemented in a software tool for realtime	Urban and rural areas	Mathematic models and real-life evaluation (qual and quant)	Geographical distances<br>Population<br>Probability of new medical emergencies<br>Ambulance stations<br>Travel times<br>Chain relocation effects EMS sleepingtimes	Response time<br>Feasibility of the software	When dispatchers worked in a consistent way, relocation decisions were made faster, dispatchers had a better overview of the	High quality

	decision support	EMS shiftworking times	available ambulances. The use of policies instead of dispatcher intuition improved efficiency.

References for included studies

1. van Barneveld TC, Bhulai S, van der Mei RD. A dynamic ambulance management model for rural areas : Computing redeployment actions for relevant performance measures. Health Care Manag Sci 2017;20(2):165-86. doi: 10.1007/s10729-015-9341-3 [published Online First: 2015/10/05]

2. Bélanger V, Kergosien Y, Ruiz A, Soriano P. An empirical comparison of relocation strategies in real-time ambulance fleet management. Comput Ind Eng. 2016;94:216-29.

3. Billhardt H, Lujak M, Sánchez-Brunete V, et al. Dynamic coordination of ambulances for emergency medical assistance services. Knowledge-Based Systems 2014;70:268-80. doi: https://doi.org/10.1016/j.knosys.2014.07.006

4. Carvalho AS, Captivo ME, Marques I. Integrating the ambulance dispatching and relocation problems to maximize system's preparedness. European Journal of Operational Research 2020;283(3):1064-80. doi: https://doi.org/10.1016/j.ejor.2019.11.056

5. Degel D, Wiesche L, Rachuba S, et al. Time-dependent ambulance allocation considering data-driven empirically required coverage. Health Care Manag Sci 2015;18(4):444-58. doi: 10.1007/s10729-014-9271-5 [published Online First: 2014/03/13]

6. Deng Y, Zhang Y, Pan J. Optimization for Locating Emergency Medical Service Facilities: A Case Study for Health Planning from China. Risk Manag Healthc Policy 2021;14:1791-802. doi: 10.2147/rmhp.S304475 [published Online First: 2021/05/11]

7. Enayati S, Mayorga ME, Rajagopalan HK, et al. Real-time ambulance redeployment approach to improve service coverage with fair and restricted workload for EMS providers. Omega 2018;79:67-80. doi: https://doi.org/10.1016/j.omega.2017.08.001

8. Hajiali M, Teimoury E, Rabiee M, et al. An interactive decision support system for real-time ambulance relocation with priority guidelines. Decision Support Systems 2022;155:113712. doi: https://doi.org/10.1016/j.dss.2021.113712

9. Jagtenberg CJ, Bhulai S, van der Mei RD. Dynamic ambulance dispatching: is the closest-idle policy always optimal? Health Care Manag Sci 2017;20(4):517-31. doi: 10.1007/s10729-016-9368-0 [published Online First: 2016/05/22]

10. Jánošíková Ľ, Jankovič P, Kvet M, et al. Coverage versus response time objectives in ambulance location. Int J Health Geogr 2021;20(1):32. doi: 10.1186/s12942-021-00285-x [published Online First: 2021/07/04]

11. Karpova Y, Villa F, Vallada E, Vecina MÁ. Heuristic algorithms based on the isochron analysis for dynamic relocation of medical emergency vehicles. Expert Syst. Appl. 2023;212:118773. Available online Sept 2022.

12. Wei Lam SS, Zhang ZC, Oh HC, et al. Reducing ambulance response times using discrete event simulation. Prehospital emergency care : official journal of the National Association of EMS Physicians and the National Association of State EMS Directors 2014;18(2):207-16. doi: 10.3109/10903127.2013.836266 [published Online First: 2013/10/19]

13. Lam SS, Zhang J, Zhang ZC, et al. Dynamic ambulance reallocation for the reduction of ambulance response times using system status management. The American journal of emergency medicine 2015;33(2):159-66. doi: 10.1016/j.ajem.2014.10.044 [published Online First: 2014/12/10]

14. Lam SSW, Ng CBL, Nguyen F, et al. Simulation-based decision support framework for dynamic ambulance redeployment in Singapore. Int J Med Inform 2017;106:37-47. doi: 10.1016/j.ijmedinf.2017.06.005 [published Online First: 2017/09/06]

15. Nogueira LC, Jr., Pinto LR, Silva PM. Reducing Emergency Medical Service response time via the reallocation of ambulance bases. Health Care Manag Sci 2016;19(1):31-42. doi: 10.1007/s10729-014-9280-4 [published Online First: 2014/04/20]

16. Roa JC, Escobar, J.W., Marín- Moreno, C.A. An online real-time matheuristic algorithm for dispatch and relocation of ambulances. International Journal of Industrial Engineering Computations 2020;11(3):443-68.

17. Schmid V. Solving the dynamic ambulance relocation and dispatching problem using approximate dynamic programming. Eur J Oper Res 2012;219(3):611-21. doi: 10.1016/j.ejor.2011.10.043 [published Online First: 2012/06/16]

18. Strauss C, Bildstein G, Efe J, et al. Optimizing Emergency Medical Service Structures Using a Rule-Based Discrete Event Simulation-A Practitioner's Point of View. Int J Environ Res Public Health 2021;18(5) doi: 10.3390/ijerph18052649 [published Online First: 2021/04/04]

19. Swalehe M, Aktas SG. Dynamic Ambulance Deployment to Reduce Ambulance Response Times Using Geographic Information Systems: A Case Study of Odunpazari District of Eskisehir Province, Turkey. Procedia Environmental Sciences 2016;36:199-206. doi: https://doi.org/10.1016/j.proenv.2016.09.033

20. Umam, M. I. H., Santosa, B., & Siswanto, N. (2022). A Simulation Optimization for Location and Allocation of Emergency Medical Service. International Int. j. online biomed. eng, 18(11), pp. 158–172.

21. van Buuren M, Jagtenberg, C., van Barneveld, T.,van der Mei, R., Bhulai, S. Ambulance Dispatch Center Pilots Proactive Relocation Policies to Enhance Effectiveness. Interfaces 2018;48(3):235-46.

BMJ Publishing Group Limited (BMJ) disclaims all liability and responsibility arising from any reliance placed on this supplemental material which has been supplied by the author(s)
