# Supplementary material for: Dynamic ambulance relocation: a scoping review
Source: BMJ Open. 2023 Dec 14;13(12):e073394. doi: 10.1136/bmjopen-2023-073394 (PMC10729233; doi:10.1136/bmjopen-2023-073394)
Supplement: Author's manuscript [file bmjopen-2023-073394.draft_revisions.pdf]

BMJ Open is committed to open peer review. As part of this commitment we make the peer review history of every article we publish publicly available.

When an article is published we post the peer reviewers' comments and the authors' responses online. We also post the versions of the paper that were used during peer review. These are the versions that the peer review comments apply to.

The versions of the paper that follow are the versions that were submitted during the peer review process. They are not the versions of record or the final published versions. They should not be cited or distributed as the published version of this manuscript.

BMJ Open is an open access journal and the full, final, typeset and author-corrected version of record of the manuscript is available on our site with no access controls, subscription charges or pay-per-view fees (http://bmjopen.bmj.com).

If you have any questions on BMJ Open's open peer review process please email info.bmjopen@bmj.com

BMJ Open

Dynamic ambulance relocation: a scoping review

Journal:	*BMJ Open*
Manuscript ID	bmjopen-2023-073394
Article Type:	Original research
Date Submitted by the Author:	06-Mar-2023
Complete List of Authors:	Becker, Julia; Institute for Disaster and Emergency Management<br>Kurland, Lisa; Örebro University; Örebro University Hospital<br>Höglund, Erik; Örebro University; Örebro Country Council, Ambulance Department<br>Hugelius, Karin; Örebro University; Örebro Country Council, Ambulance Department
Keywords:	ACCIDENT & EMERGENCY MEDICINE, Organisation of health services < HEALTH SERVICES ADMINISTRATION & MANAGEMENT, Systematic Review

SCHOLARONE™
Manuscripts

I, the Submitting Author has the right to grant and does grant on behalf of all authors of the Work (as defined in the below author licence), an exclusive licence and/or a non-exclusive licence for contributions from authors who are: i) UK Crown employees; ii) where BMJ has agreed a CC-BY licence shall apply, and/or iii) in accordance with the terms applicable for US Federal Government officers or employees acting as part of their official duties; on a worldwide, perpetual, irrevocable, royalty-free basis to BMJ Publishing Group Ltd ("BMJ") its licensees and where the relevant Journal is co-owned by BMJ to the co-owners of the Journal, to publish the Work in this journal and any other BMJ products and to exploit all rights, as set out in our licence.

The Submitting Author accepts and understands that any supply made under these terms is made by BMJ to the Submitting Author unless you are acting as an employee on behalf of your employer or a postgraduate student of an affiliated institution which is paying any applicable article publishing charge ("APC") for Open Access articles. Where the Submitting Author wishes to make the Work available on an Open Access basis (and intends to pay the relevant APC), the terms of reuse of such Open Access shall be governed by a Creative Commons licence – details of these licences and which Creative Commons licence will apply to this Work are set out in our licence referred to above.

Other than as permitted in any relevant BMJ Author's Self Archiving Policies, I confirm this Work has not been accepted for publication elsewhere, is not being considered for publication elsewhere and does not duplicate material already published. I confirm all authors consent to publication of this Work and authorise the granting of this licence.

For peer review only

Dynamic ambulance relocation: a scoping review

Authors:

Julia Becker[1], Lisa Kurland [2,3,4] Erik Höglund [2,4], Karin Hugelius [2,4]

1. Institute for Disaster and Emergency Management, Berlin, Germany
2. Faculty of Medicine and Health, Örebro University, Örebro, Sweden
3. Emergency Department, Örebro University Hospital, Örebro, Sweden
4. Ambulance Department, Örebro Country Council, Örebro, Sweden

* Corresponding author

Corresponding author contacts:

Karin Hugelius
E-mail: karin.hugelius@oru.se
Phone: +46 70 219 59 35

Word count: 2908

For peer review only

2

ABSTRACT

Objectives: Dynamic ambulance relocation means that the operators at a dispatch centre place an ambulance in a temporary location, with the goal of optimising coverage and response times in future medical emergencies. The aim of this study was to scope the current research on dynamic ambulance relocation.

Design: A scoping review was conducted using a structured search in PubMed, Scopus and Web of Science. In total, 18 papers were included. PRISMA Extension for Scoping Reviews was followed.

Results: Most papers described research with experimental designs involving the use of mathematical models to calculate the optimal use and temporary relocations of ambulances. The models relied on several variables, including distances, locations of hospitals, demographic-geological data, estimation of new emergencies, emergency medical services (EMS) working hours and other data. Some studies used historic ambulance dispatching data to develop models. Only one study reported a prospective, real-time evaluation of the models and the development of technical systems. No study reported on either positive or negative medical outcomes or real-life chain effects from the dynamic relocation of ambulances.

Conclusions: Current knowledge on dynamic relocation is dominated by mathematical and technical support data that have calculated optimal locations of ambulance services. By contrast, knowledge of how patient outcomes and the working environment are affected by dynamic ambulance dispatching is lacking. This review has highlighted several gaps in the scientific coverage of the topic. The primary concern is the lack of studies reporting on patient outcomes, and the limited knowledge regarding several key factors, including the optimal use of ambulances in rural areas, turnaround times, domino effects and aspects of working environment for EMS personnel. Therefore, addressing these knowledge gaps is important in future studies.

ARTICLE SUMMERY

Strengths and limitations of this study

- This scoping review systematically summarises the scientific knowledge on dynamic relocation dispatching of ambulances published in both medical and technological papers.
- It identifies gaps in the existing literature and suggests that studies on patient outcomes following dynamic relocation of ambulances are strongly needed.
- Limitations of this review are that the systematic search was conducted in three databases and included a limited number of papers reporting on dynamic relocation of ambulances from non-experimental settings and rural areas.

BACKGROUND

Emergency medical services (EMS) are an essential part of the emergency care system. An increasing number of emergency calls and EMS use, and the resultant influx into the emergency department, are driving the need for optimal use of existing resources [1]. For this reason, considerable research has been devoted to optimising the use of the EMS from a dispatching and logistical perspective [2].

Dispatching ambulances is a complex task, as dispatch decisions include both strategic and operational considerations, such as the number and location of fixed ambulance stations and how the available ambulances should be used to optimise ambulance coverage[2]. The responsiveness of the EMS depends on both stable factors, such as the location of a hospital, and factors that are

For peer review only

constantly changing, such as traffic situations or weather conditions [3]. Two main models have been identified for operational decision-making on how best to use available resources and which ambulance to assign to a specific emergency call. The first model is the static location model (i.e. the ambulance is dispatched to a specific/given ambulance station and it returns to its designated ambulance station after a completed mission). The second model is dynamic relocation [2 4], also termed dynamic allocation or dynamic ambulance deployment. Dynamic relocation of ambulance vehicles means that the operator at the dispatch centre directs the ambulance to a temporary location (i.e. a location other than the regular, static ambulance station) with the purpose of reducing the response time. In this review, the term dynamic ambulance relocation was used to describe this kind of flexible repositioning of available ambulance resources. The use of dynamic ambulance relocation in Sweden varies, from not used at all to over 20 relocation missions per 1000 inhabitants[5]. In the Örebro County Council (Sweden), the number of dynamic ambulance relocation missions increased from 2% in 2021 to over 5% in 2022 [6].

Many studies have focused on technical solutions or relied on artificial intelligence to support ambulance dispatch operators in allocating the ambulance to the 'best' location [7]. Most of the focus has been on time. Prehospital time is a crucial factor for the survival of critically ill or injured patients [8 9]. In particular, response time (e.g. the time from dispatch until the ambulance is at the patient's side)[10] and turnaround time (e.g. the time from being dispatched until the ambulance is ready for new assignments) seems to be critial[11]. However, little is still known about the dispatch model and prehospital time, and no consensus has been reached regarding the most effective model for ambulance dispatching. The increased burden on the EMS system and the considerable variation in the use of dynamic ambulance relocation have created a need to summarise the current scientific knowledge on dynamic ambulance relocation and to identify knowledge gaps in the field. Therefore, the aim of this review is to scope the current research on dynamic ambulance relocation.

METHODS

A scoping review was conducted using the Arksey & O'Malley methodology [12]. The PRISMA Extension for Scoping Reviews (PRISMA-ScR) protocol was followed and used to report the study.

Stage 1: Identifying the research question
The research question for this study was 'What is known about dynamic ambulance allocations?'

Stage 2: Identifying relevant studies (search strategy)
Two structured searches were performed in the PubMed database, Scopus and Web of Science by one author (KH), with support from an academic librarian. The search terms used and the number of records for the searches are shown in Table 1. The database searches were conducted on October 27 and December 09, 2022. Both searches were limited to original papers published in English between 2012 and 2022. In addition, a manual search in Google Scholar was performed by the third author (EH), which retrieved two additional papers.

Stage 3: Study selection
The study selection process was conducted using Covidence software (Covidence systematic review software, Veritas Health Innovation, Melbourne, Australia). All papers identified in the literature search were exported to the Covidence systematic review software, where duplications were sought and removed. Thereafter, all records were screened by the first and second authors (JB and KH) by reading the title and abstract. For inclusion, an original research paper had to (1) report on dynamic ambulance allocation for road ambulances and (2) be published between 2013 and 2022. Both qualitative and quantitative studies were included. Papers reporting on air ambulance services, fixed locations of ambulance resources (such as the optimal location of an ambulance station), review

For peer review only - http://bmjopen.bmj.com/site/about/guidelines.xhtml

studies, case studies or editorial texts were excluded. The full-text review assessed the entire paper and its eligibility for the study. In this stage of the selection process, the reason for exclusion was documented (Figure 1).

In addition to the original scoping review methodology, a quality appraisal was performed [13], using the Critical Appraisal Skills Programme (CASPS) checklist for cohort studies[14]. The quality appraisal was performed by the first author (JB) and validated by the fourth author (KH) (see Supplementary Material).

Stage 4: Charting the data

All included studies were reviewed, and the following information was extracted: author(s) and year of publication, study location, design, aim, focus/ aim of the study, setting (urban and/or rural areas), type of modelling, variables used, main findings and quality appraisal.

Stage 5: Collating, summarising and reporting the results.

The results are presented below.

Patient and Public Involvement statement

No patients were included in the study

Table 1: Overview of the search strategy

	Search terms	Number of records
PubMed*	S1: [ambulance dispatch]	1089
	S2: [dynamic]	985 250
	S4: [relocation]	15779
	S3: [ambulance dispatch] AND [dynamic]	29
	S5: [ambulance dispatch] AND [relocation]	5
	Total: S3+ S5	34
Web of Science*	S1: [ambulance dispatch]	679
	S2: [dynamic]	55 826
	S3: [dynamic ambulance dispatching]	36
	Total: S3	48
Scopus**	S1: [ambulance dispatch]	645
	S2: [dynamic]	6785
	S3: [dynamic ambulance dispatching]	71
	Total: S3	71
	Total exported to Covidence	94
After removing duplications		48
* Date of the search: October 27, 2022, **Date for search: December 09, 2022.		

Insert Figure 1 about here

Figure 1: Overview of the selection process and reasons for exclusion.

For peer review only

5

RESULTS

In total, 18 original papers were included in this scoping review. The included articles were published between 2014 and 2022, and the studies were conducted in Austria (n=1), Brazil (n=1), China (n=1), Colombia (n=1), Germany (n=1), Iran (n=2), Portugal (n=1), Singapore (n=3), Slovakia (n=1), Switzerland (n=1), the Netherlands (n=3), Turkey (n=1) and the USA (n=1). The studies were assessed as medium (n=13) or high (n=5) in quality (see Table 2, supplementary material).

Study contexts and designs

The studies were most often conducted in urban areas, including metropolitan cities. Few studies were conducted in rural areas (see Table 3). One study [15] was only based on emergency calls for critical patients.

All studies were based on mathematical simulation models, with a large variation of outcome variables. Most studies (n=12) used an experimental design and simulations based on retrospective EMS data and medical emergencies within a specified region (see Table 3). Three studies used experimental design (theoretical applications with no simulation) and two studies reported a prospective, real-time evaluation of the models and technical systems (see Table 2, supplementary material). Two studies evaluated their developed models in real-time evaluations, one in both urban and rural areas [16] and one in an urban context [17].

Outcome variables and variables used in predictions of optimal ambulance dispatching and/ or relocation

Most studies (n=14) used response time as the primary outcome variable for the models developed and/ or tested. The information used to develop the mathematical models and/or computer-based programs was collected from retrospective data from real EMS activities (n=6), geographic and demographic data from national databases or Google Earth (n=6), estimations made by the research teams (n=3) or a combination of these sources within the same study (n=3) (see Table 3). Some studies did not specify the sources for their variable data (n=5). All studies included three or more variables in their models.

Table 3. Summary of included studies

		Number of studies N= 18
**Study design**	Experimental design and simulations based on retrospective EMS data	12
	Experimental design with no simulation/ testing	3
	Experimental design with real-time evaluation of models	2
**Data used**	Retrospective data from EMS activities	4
	Geographic and demographic data from national databases or Google Earth	6
	Estimations made by the research teams	3
	Combinations of estimations and official data	3
**Study context**	Urban areas	12
	Rural areas	2
	Both urban and rural areas	3
**Outcome variable**	Response time	14

For peer review only

	Turnaround time	3
	Workload on each ambulance	1
	Availability for new calls	2
	Perceived feasibility of software	1
**Variables used in the models**	Travel times and road network	14
	Current location of ambulances	11
	Number of available ambulances	7
	Location of hospitals	5
	Population living in a defined geographical area	4
	Time of day	4
	Response time	5
	Expected occurrence of medical emergencies within a specified area or timeframe	5
	Type of emergency or priority of the call	3
	Turnaround time	2
	Time at scene	2
	Uncovered emergencies	1
	Economical costs	1
	EMS working hours	3
	EMS workload	2
	Chain relocation effects	1

Outcomes from dynamic ambulance relocation

No study reported or discussed medical outcomes or clinical effects of dynamic relocation of ambulances. Three studies reported decreased response times when using dynamic relocation compared to ordinary dispatching and when using ordinary ambulance stations. Reported time saved was 44 seconds [18], 54 seconds [19] and undefined saving of time [17]. No study reported on changes in response time or turnaround time in rural areas. Chain effects on other emergency calls due to dynamic ambulance relocations were reported[16]. One study reported that dynamic ambulance relocation reduced the total working time of all ambulances by about 9% per shift [17].

DISCUSSION

This review showed that most studies on dynamic ambulance relocation were based on experimental study designs and presented mathematical models in which three or more variables were used to calculate the optimal dynamic relocation of an ambulance with respect to response time. Most of the variables used in the models were fixed, and few studies evaluated the models or programs in prospective, real-life settings. Most studies were conducted in rural areas. No study reported on medical benefits or patient outcomes attained by the use of dynamic ambulance relocation.

Ambulance dispatching and the optimal use and location of ambulances are important but challenging questions to analyse. Most variables used in the models presented were static, gathered from GIS systems or similar techniques. New techniques, such as artificial intelligence (AI), may offer additional tools for decision-making and dispatching of ambulances in the future [20]. However, dispatchers also use other variables, such as medical priority, type of medical care required, clinical judgement and their own professional experience, when making decisions on ambulance dispatching [21]. Therefore, an important consideration is to identify how fixed variables can be incorporated into decision-making systems, while also recognising the limitations of these tools.

No consensus has yet been reached on how to assess, define or measure the quality of care within the prehospital setting[22]; however, prehospital response times have long been a core component in

For peer review only

these discussions [10]. Most of the studies considered in this review used response time as the primary outcome. However, trauma has traditionally been used as a role model for time-critical medical conditions, as prolonged response time is associated with increased mortality in trauma victims [23], especially in rural areas [11]. Other studies suggest that prolonged prehospital time may have positive, negative and neutral associations with trauma patient mortality [24]. However, the actual time gained by using dynamic ambulance relocations, in comparison to static ambulance allocation (e.g. the ordinary ambulance station), was only studied in two of the articles, and in those, the time saved was less than a minute. Hence, whether this time saved had clinical relevance (i.e. affected the outcome of the patients) cannot be determined.

This review also does not allow any judgement of the value of dynamic relocation based on turnaround times, as only two of the included studies reported turnaround times. Both of these studies were conducted in large cities with many available emergency departments and neither compared turnaround times when dispatching using fixed ambulance locations versus dynamic relocation. Dynamic ambulance relocations may also cause a chain reaction, as temporary relocation of one ambulance may lead to a lack of resources in the original location, thus leading to new relocations and so on. These effects were covered in only one of the included studies. Therefore, no conclusions can be drawn regarding reductions in the response or turnaround time by dynamic ambulance relocation based on the published studies.

Most studies developed and evaluated models based on EMS in urban areas. Notable differences exist between urban and rural areas regarding ambulance coverage, and turnaround time is most likely shorter in a city than in rural areas. A shortage of ambulance resources may therefore be more critical in rural areas than in urban areas. If the number of ambulances is small, the effect of a shortage or temporary relocation of one ambulance is more noticeable than in a city with more ambulances per area [25]. Conversely, the statistical risk for the occurrence of a time-critical condition is considerably lower in rural areas with small populations than in cities with large populations. However, the distances might be longer in rural areas than in urban areas. Even a temporary relocation may cause a significant delay in EMS response time; therefore, the effects of dynamic relocation might be more prominent in rural areas. Given the vulnerability of EMS coverage in rural areas, dynamic ambulance relocation in these areas needs to be prioritised in future studies.

The well-being and working environment of EMS personnel is another relevant aspect of the optimal use of available ambulances. Long working hours, shift work and short times between the missions used for recovery are associated with work-related stress and mental health problems [26]. A practical consequence of dynamic ambulance relocation can be limited access to a toilet, kitchen or resting facilities for ambulance personnel. In this review, only one model included variables associated with the ambulance personnel working times, shifts and night rest. Only one study reported on workload effects. Working hours, working environment and workload are all important for both the effectiveness and efficiency of the EMS and should be considered when creating models for dynamic ambulance relocations.

Limitations

This scoping review has several limitations. One was that the search was performed in three databases. However, these provided a broad selection of medical and non-medical journals and were therefore considered sufficient. By including studies from medical, technical and mathematical disciplines, the format for paper presentations varied. According to the scoping review methodology, only data from the results section should be charted and used in the review results. However, since the structure of the included papers varied, valuable information or outcomes stated under other

For peer review only - http://bmjopen.bmj.com/site/about/guidelines.xhtml

8

headings were also extracted and included in the results of this review if the data or outcome referred to the actual study. A quality appraisal is not mandatory when using the scoping review methodology. However, to judge the value of the findings, a quality appraisal was found to add value and was therefore conducted.

CONCLUSION

The current knowledge on dynamic relocation emanates from mathematical and technical support used to calculate the optimal locations of ambulance services. However, knowledge of how patient outcomes and working environments are affected by dynamic ambulance dispatching is lacking. This review has highlighted several gaps in the scientific coverage of the topic. The primary concerns are the lack of studies reporting on patient outcomes and the limited knowledge about several key factors, including the optimal use of ambulances in rural areas, turnaround times, chain effects and aspects of working environment for EMS personnel. Therefore, addressing these knowledge gaps will be important in future studies.

List of abbreviations

Emergency Medical Services (EMS)

PRISMA Extension for Scoping Reviews (PRISMA-ScR)

Funding

This research received no specific grant from any funding agency in the public, commercial or not-for-profit sectors.

DECLARATIONS

Ethics approval and consent to participate
This study does not involve participants; therefore, ethical approval or consent was not required.

Availability of data and materials
Data sharing was not applicable, as no data sets were generated and/or analysed in this study.

Competing interests
The authors declare that they have no competing interests.

Author contributions
LB: Conceptualization, data collection, analysis, writing of the manuscript LK: Conceptualization, analysis, writing of the manuscript, EH: Conceptualization, data collection, confirming analysis, writing of the manuscript, KH: Conceptualization, data collection, analysis, writing and editing of the manuscript. All authors read and approved the final manuscript.

References

1. Andrew E, Nehme Z, Cameron P, *et al.* Drivers of increasing emergency ambulance demand. *Prehosp Emerg Care* 2020;24(3):385-93. doi: 10.1080/10903127.2019.1635670 [published Online First: 2019/06/27]
2. Bélanger V, Ruiz A, Soriano P. Recent optimization models and trends in location, relocation, and dispatching of emergency medical vehicles. *Eur J Oper Res* 2019;272(1):1-23. doi: 10.1016/j.ejor.2018.02.055

For peer review only - http://bmjopen.bmj.com/site/about/guidelines.xhtml

9

3. Jánošíková L, Jankovič P, Kvet M, *et al.* Reorganization of an emergency medical system in a mixed urban-rural area. *Int J Environ Res Public Health* 2022;19(19):12369. doi: 10.3390/ijerph191912369 [published Online First: 2022/10/15]
4. Schmid V, Doerner KF. Ambulance location and relocation problems with time-dependent travel times. *Eur J Oper Res* 2010;207(3):1293-303. doi: 10.1016/j.ejor.2010.06.033
5. Nysam. Ambulanssjukvård. Nyckeltal 2019. Rapport Maj 2020. (In English: Ambulance care. Statistics 2019. Report May 2020). Stockholm, 2020. Available at: https://nysam.com/images/Rapporter/Ambulanssjukvard_2019_Nysamrapport.pdf
6. Örebro County Council AD. Statistics. Örebro, Sweden, 2023.
7. Neira-Rodado D, Escobar-Velasquez JW, McClean S. Ambulances deployment problems: categorization, evolution and dynamic problems review. *ISPRS Int J Geo-Inf* 2022;11(2):109. doi: 10.3390/ijgi11020109
8. Chen X, Guyette FX, Peitzman AB, *et al.* Identifying patients with time-sensitive injuries: Association of mortality with increasing prehospital time. *J Trauma Acute Care Surg* 2019;86(6):1015-22. doi: 10.1097/ta.0000000000002251 [published Online First: 2019/05/28]
9. van Rein EAJ, van der Sluijs R, Houwert RM, *et al.* Effectiveness of prehospital trauma triage systems in selecting severely injured patients: Is comparative analysis possible? *Am J Emerg Med* 2018;36(6):1060-69. doi: 10.1016/j.ajem.2018.01.055 [published Online First: 2018/02/06]
10. El Sayed MJ. Measuring quality in emergency medical services: a review of clinical performance indicators. *Emerg Med Int* 2012;2012:161630. doi: 10.1155/2012/161630 [published Online First: 2011/11/03]
11. Harmsen AM, Giannakopoulos GF, Moerbeek PR, *et al.* The influence of prehospital time on trauma patients outcome: a systematic review. *Injury* 2015;46(4):602-9. doi: 10.1016/j.injury.2015.01.008 [published Online First: 2015/01/30]
12. Arksey H, O'Malley, L. Scoping studies: towards a methodological framework. *Int J Soc Res Methodol: Theory & Pract* 2005;8(1):19-32. doi: 10.1080/1364557032000119616
13. Sucharew H, Macaluso M. Progress notes: methods for research evidence synthesis: the scoping review approach. *J Hosp Med* 2019;14(7):416-18. doi: 10.12788/jhm.3248 [published Online First: 2019/06/30]
14. (CASP) CASP. CASP Coort Study Checklists Available at: https://casp-uk.net/casp-tools-checklists/: CASP; 2020 [accessed November 04 2020].
15. Billhardt H, Lujak M, Sánchez-Brunete V, *et al.* Dynamic coordination of ambulances for emergency medical assistance services. *Knowl Based Syst* 2014;70:268-80. doi: 10.1016/j.knosys.2014.07.006
16. van Buuren M, Jagtenberg, C., van Barneveld, T.,van der Mei, R., Bhulai, S. Ambulance dispatch center pilots proactive relocation policies to enhance effectiveness. *Interfaces* 2018;48(3):235-46. doi: 10.1287/inte.2017.0936
17. Hajiali M, Teimoury E, Rabiee M, *et al.* An interactive decision support system for real-time ambulance relocation with priority guidelines. *Decis Support Syst* 2022;155:113712. doi: 10.1016/j.dss.2021.113712
18. Lam SS, Zhang J, Zhang ZC, *et al.* Dynamic ambulance reallocation for the reduction of ambulance response times using system status management. *Am J Emerg Med* 2015;33(2):159-66. doi: 10.1016/j.ajem.2014.10.044 [published Online First: 2014/12/10]
19. Deng Y, Zhang Y, Pan J. Optimization for locating emergency medical service facilities: a case study for health planning from China. *Risk Manag Healthc Policy* 2021;14:1791-802. doi: 10.2147/rmhp.S304475 [published Online First: 2021/05/11]
20. Ceklic E, Ball S, Finn J, *et al.* Ambulance dispatch prioritisation for traffic crashes using machine learning: A natural language approach. *Int J Med Inform* 2022;168:104886. doi: 10.1016/j.ijmedinf.2022.104886 [published Online First: 2022/10/29]

For peer review only

10

21. Hosseini SMR, Maleki M, Gorji HA, *et al.* Factors affecting emergency medical dispatchers' decision-making: a qualitative study. *J Multidiscip Healthc* 2018;11:391-98. doi: 10.2147/jmdh.S159593 [published Online First: 2018/09/04]
22. Rehn M, Kruger AJ. Quality improvement in pre-hospital critical care: increased value through research and publication. *Scand J Trauma, Resusc Emerg Med* 2014;22:34. doi: 10.1186/1757-7241-22-34 [published Online First: 2014/06/03]
23. Brown JB, Rosengart MR, Forsythe RM, *et al*. Not all prehospital time is equal: Influence of scene time on mortality. *J Trauma Acute Care Surg* 2016;81(1):93-100. doi: 10.1097/ta.0000000000000999 [published Online First: 2016/02/18]
24. Bedard AF, Mata LV, Dymond C, *et al*. A scoping review of worldwide studies evaluating the effects of prehospital time on trauma outcomes. *Int J Emerg Med* 2020;13(1):64. doi: 10.1186/s12245-020-00324-7 [published Online First: 2020/12/11]
25. van Barneveld TC, Bhulai S, van der Mei RD. A dynamic ambulance management model for rural areas : Computing redeployment actions for relevant performance measures. *Health Care Manag Sci* 2017;20(2):165-86. doi: 10.1007/s10729-015-9341-3 [published Online First: 2015/10/05]
26. Lawn S, Roberts L, Willis E, *et al.* The effects of emergency medical service work on the psychological, physical, and social well-being of ambulance personnel: a systematic review of qualitative research. *BMC Psychiatry* 2020;20(1):348. doi: 10.1186/s12888-020-02752-4 [published Online First: 2020/07/06]

For peer review only

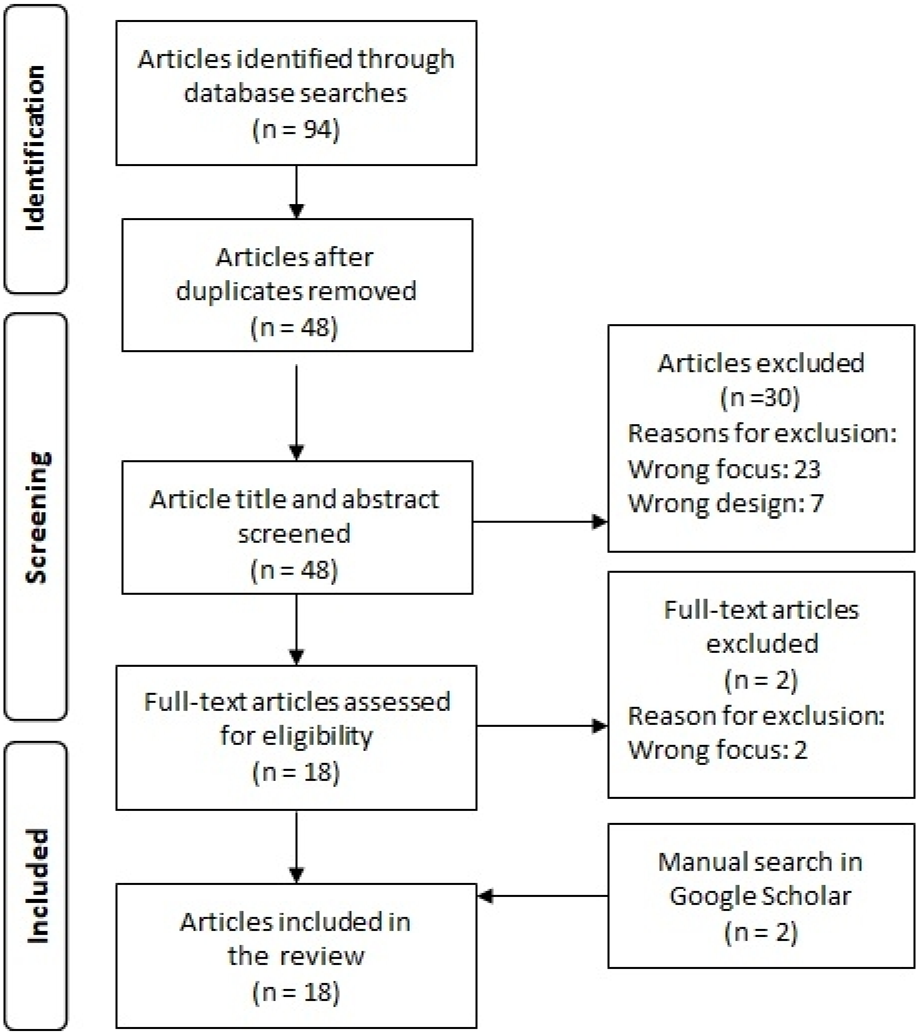

Overview of the selection process and reasons for exclusion.

124x135mm (96 x 96 DPI)

Supplementary material 1: Quality appraisal according to CASP protocols

Author/ year	Did the study address a clearly focused issue?	Was the cohort requited in an acceptable way?	Was the exposure accurately measured to minimize bias?	Was the outcome accurately measured to minimize bias?	Have the authors identified all important confounding factors?	Have they taken account of the confounding factors in the design and/or analysis?	Was the follow-up of subjects complete and long enough?	How precise are the results?	Do you believe in the results?	Can the results be applied to the local population?	Do the results of this study fit with other available evidence?	Overall quality
Barneveldt 2017	Yes	N/A	N/A	Some	Some	Some	Not able to judge	Some	Some	Yes	Yes	Medium
Billhart et al 2014	Yes	N/A	N/A	Some	No	Some	Yes	Some	Yes	Yes	Yes	Medium
Carvallo et al 2020	Yes	N/A	N/A	Some	Some	Yes	Yes	Some	Yes	Yes	Yes	High
Degel et al 2015	Yes	N/A	N/A	Some	Some	Yes	Yes	Some	Yes	Yes	Yes	Medium
Deng et al 2021	Yes	N/A	N/A	Some	Some	Some	N/A	Some	Some	Unclear	Yes	Medium
Enayati et al 2018	Yes	N/A	N/A	Some	Some	Some	Yes	Some	Some	Yes	Yes	Medium
Hajiali et al 2022	Yes	N/A	N/A	Yes	Some	Yes	No	Good	Some	Yes	Some	High
Jagtenberg et al 2017	Yes	N/A	N/A	Some	Some	Yes	N/A	Some	Some	Unclear	Yes	Medium
Jánošíková et al 2021	Yes	N/A	N/A	Some	Some	Some	Not able to judge	Some	Some	Yes	Yes	Medium
Lam et al 2014	Yes	N/A	N/A	Yes	Yes	Some	Yes	Good	Yes	Yes	Yes	High
Lam et al 2015	Yes	N/A	N/A	Yes	Some	Some	Yes	Some	Yes	Yes	Yes	Medium
Lam et al 2017	Yes	N/A	N/A	Yes	Yes	Yes	Yes	Some	Yes	Yes	Yes	High
Nogueira et al 2016	Yes	N/A	N/A	Some	Some	Some	Some	Some	Some	Yes	Yes	Medium
Roa et al 2020	Yes	N/A	N/A	Some	Some	Some	Some	Some	Some	Yes	Yes	Medium
Schmid 2021	Yes	N/A	N/A	Some	Some	Some	Yes	Some	Some	Yes	Yes	Medium
Strauss et al 2021	Yes	N/A	N/A	Some	Some	Some	Unclear	Some	Yes	Yes	Yes	Medium
Swalehe & Aktas 2016	Yes	N/A	N/A	Some	Some	Some	Unclear	Some	Yes	Yes	Yes	Medium
Van Buuren et al 2018	Yes	N/A	N/A	Yes	Some	Yes	Yes	Good	Yes	Yes	Yes	High

For peer review only

Table 2: Overview of included studies

Authors and publication year	Study location	Design	Aim of the study	Setting	Type of modelling	Variables used in the model	Outcome variable	Main findings	Quality appraisal
Barneveldt et al 2017[1]	The Netherlands	Experimental	To develop a method to obtain good relocation strategies in real time	Rural areas	Mathematic models and scenario probabilities based on retrospective data	Demand locations<br>Time of the day<br>Level of priority<br>Total number of ambulances<br>Treatment time on scene	Response time	A one-step look-ahead heuristic model that relocated ambulances to minimize the response time was presented	Medium quality
Billhardt et al 2014[2]	Spain	Experimental	To present an event-driven system for dynamic ambulance assignment and redeployment	Urban areas	Mathematic models and scenario probabilities	Geographical location<br>Available ambulances<br>(The study only included critical patients)	Response time	Mathematic models can reduce the response times for EMS services. However, more variables need to be considered and included in such models	Medium quality
Carvalho et al 2020[3]	Portugal	Experimental with simulation	To develop models to solve ambulance dispatching and relocation problems	Urban areas	Mathematic models and scenario probabilities based on retrospective data (n=20 000)	Available ambulances<br>Travel times<br>Ambulance stations locations<br>Dynamic relocation spots<br>Waiting time<br>Maximum response times<br>Uncovered emergencies	Availability for new calls	A mixed-integrated programming model and an experimental model was presented. Both approaches used a time preparedness measure to evaluate the	High quality

For peer review only

								system’s capability to handle new emergencies	
Degel et al 2015[4]	Germany	Experimental with simulation	To support decision on location planning for ambulances	Rural areas	Mathematic models and scenario probabilities based on retrospective data	Travel time<br>Required coverage<br>Variation in fleet<br>Flexible locations	Availability for new calls	The inclusion of time-dependent and empirical data had a substantial impact on the solution	Medium quality
Deng et al 2021[5]	China	Experimental	To optimize the EMS system	No information given	Mathematic models	Population density<br>Road network<br>Location of hospitals<br>Location of flexible locations	Response time	The solution presented could effectively achieve a population coverage within 15 minutes, with a minimum number of temporary ambulance points in addition to existing ambulance stations	Medium quality
Enayati et al 2018[6]	USA	Experimental with simulation	To develop a real-time model to maximize coverage with minimum possible total travel time	Urban	Mathematic models and scenario probabilities based on retrospective data (n=50 000)	Initial fleet size of ambulances<br>Travel limit (max 20 min)<br>Maximum allowed workload of each ambulance<br>Day and time<br>Shift times (12h)	Turnaround time	The modeling of the real time deployment model resulted in improvement in average coverage and prevented accumulated workload for	Medium quality

For peer review only

								ambulance personnel	
Hajiali et al, 2022[7]	Iran	Experimental	To develop a decision support system for optimal ambulance coverage	Urban	Mathematic models and real-life evaluation (1 week) of response time and workload for each ambulance	Base location Demand zones and time ranges from these (7 min, 15 min, 25 min) Number of available ambulances Relocation time Working shift time (12h)	Response time Workload	The decision support system decreased response times and reduced the total working time of all ambulances by about 9% per shift.	Hight quality
Jagtenberg et al 2017[8]	The Netherlands	Experimental with simulation	To present different dispatch rules and suggest a heuristic method for ambulance dispatching	Urban areas	Mathematic models and scenario probabilities	Ambulance station locations Demand locations Hospital locations the base location Time of the day Driving time	Response time	Two methods to obtain ambulance dispatch policies were reported. Their effects could not be defined	Medium quality
Jánošíková et al 2021[9]	Slovakia	Experimental with simulation	To identify optimization criterion of the EMS	Urban and rural areas	Mathematic models and scenario probabilities	Demand zones Population (and age profile) Temporary locations Travel time	Response time	The EMS systems should be designed to minimize response times, and not to maximize the number of calls served within a given time limit	Medium quality
Lam et al 2014[10]	Singapore	Experimental with simulation	To develop a discrete-event simulation	Urban area	Mathematic models and scenario probabilities	Geographical information systems (GIS) data, Travel time	Response time	Response times were improved via a more effective	High quality

			model for the Singapore EMS		based on retrospective data (n=54 548)	Ambulance location		reallocation of ambulances and dispatch policy, without additional ambulances	
Lam et al 2015[11]	Singapore	Experimental with simulation	To develop efficient ambulance allocation plans in Singapore and to evaluate its performance using the DES model.	Urban area	Mathematic models and scenario probabilities based on retrospective data (n= 52 512)	Call times Dispatch times, Scene arrival times, Hospital conveyance times Turnaround time, Locations of incident	Response time	Geographical information system-based plans reduced the median response times with 13 to 44 seconds compared to a static reallocation policy	Medium
Lam et al 2017[12]	Singapore	Experimental with simulation	To report on the previously reported work by leveraging on the DES model	Urban area	Mathematic models and scenario probabilities based on retrospective data (n=217 000)	Geographical coverage Response Times Turnaround times and utilization rates	Turnaround time	The integration of dynamic redeployments optimized the ambulance coverage	High quality
Nogueira et al 2016[13]	Brazil	Experimental with simulation	To analyze the EMS using two modeling techniques: optimization and simulation	Urban areas	Mathematic models and scenario probabilities	Ambulance station location Demand posts Hospital locations Cost for each ambulance	Response time	A balanced number of ambulances allocated across certain bases at certain times improved the system performance without	Medium quality

For peer review only

								increasing the total number of ambulances	
Roa et al 2020[14]	Colombia	Experimental with simulation	To design an algorithm to support the real-time operation of ambulances	Urban areas	Mathematic models and scenario probabilities based on retrospective data	Types of emergency, Types of ambulances Demand points Locations for ambulances Modelled on-site attention time Travel times	Response time Availability for new calls	The real-time management of ambulance fleets involved challenging decisions due to frequent, complex, and random changes in system conditions	Medium quality
Schmid 2021[15]	Austria	Experimental with simulation	To propose a stochastic dynamic model for the ambulance relocation and dispatching problem	Urban area	Mathematic models and scenario probabilities based on retrospective data (n=3748)	Location of hospitals and patients, response times, travel times, using data.	Response time	By deviating from the traditional rule of dispatching the closest ambulance available and relocating them to their home base after having finished serving, the coverage can be improved	Medium quality
Strauss et al 2021[16]	Switzerland and Germany	Experimental with simulation	To explain the major features of "rule-based discrete event simulation"	Urban and rural areas	Mathematic models and scenario probabilities	Response times, demands per day, travel time, dispatch priority	Response time	Rule-based DES models can improve regional emergency services' efficiency without increasing cost.	Medium quality

For peer review only

								All simulation-based methods suggest normative solutions and optimize EMS' performance within given healthcare system structures	
Swalehe & Aktas 2016[17]	Turkey	Experimental	To find optimal ambulance locations (stations and temporary demand points)	Urban	Computer based systems (one with information on retrospective emergency calls	Time of day<br>Population<br>Driving time<br>Road network data	Response time	Ambulance response time can be reduced with a dynamic relocation plan taking time of the day in consideration.<br>Dynamic ambulance deployment is a more effective ambulance deployment strategy than static ambulance deployment	Medium quality
Van Buuren et al 2018[18]	The Netherlands	Experimental real-life testing	To evaluate two dynamic relocation policies for operational use and implemented in a software	Urban and rural areas	Mathematic models and real-life evaluation (qual and quant)	Geographical distances<br>Population<br>Probability of new medical emergencies<br>Ambulance stations<br>Travel times	Response time<br>Feasability of the software	When dispatchers worked in a consistent way, relocation decisions were made faster, dispatchers	High quality

For peer review only

	tool for realtime decision support	Chain relocation effects EMS sleepingtimes EMS shiftworking times	had a better overview of the available ambulances. The use of policies instead of dispatcher intuition improved efficiency.

References for included studies

1. van Barneveld TC, Bhulai S, van der Mei RD. A dynamic ambulance management model for rural areas : Computing redeployment actions for relevant performance measures. Health Care Manag Sci 2017;20(2):165-86. doi: 10.1007/s10729-015-9341-3 [published Online First: 2015/10/05]

2. Billhardt H, Lujak M, Sánchez-Brunete V, et al. Dynamic coordination of ambulances for emergency medical assistance services. Knowledge-Based Systems 2014;70:268-80. doi: https://doi.org/10.1016/j.knosys.2014.07.006

3. Carvalho AS, Captivo ME, Marques I. Integrating the ambulance dispatching and relocation problems to maximize system's preparedness. European Journal of Operational Research 2020;283(3):1064-80. doi: https://doi.org/10.1016/j.ejor.2019.11.056

4. Degel D, Wiesche L, Rachuba S, et al. Time-dependent ambulance allocation considering data-driven empirically required coverage. Health Care Manag Sci 2015;18(4):444-58. doi: 10.1007/s10729-014-9271-5 [published Online First: 2014/03/13]

5. Deng Y, Zhang Y, Pan J. Optimization for Locating Emergency Medical Service Facilities: A Case Study for Health Planning from China. Risk Manag Healthc Policy 2021;14:1791-802. doi: 10.2147/rmhp.S304475 [published Online First: 2021/05/11]

6. Enayati S, Mayorga ME, Rajagopalan HK, et al. Real-time ambulance redeployment approach to improve service coverage with fair and restricted workload for EMS providers. Omega 2018;79:67-80. doi: https://doi.org/10.1016/j.omega.2017.08.001

7. Hajiali M, Teimoury E, Rabiee M, et al. An interactive decision support system for real-time ambulance relocation with priority guidelines. Decision Support Systems 2022;155:113712. doi: https://doi.org/10.1016/j.dss.2021.113712

8. Jagtenberg CJ, Bhulai S, van der Mei RD. Dynamic ambulance dispatching: is the closest-idle policy always optimal? Health Care Manag Sci 2017;20(4):517-31. doi: 10.1007/s10729-016-9368-0 [published Online First: 2016/05/22]

For peer review only

9. Jánošíková Ľ, Jankovič P, Kvet M, et al. Coverage versus response time objectives in ambulance location. Int J Health Geogr 2021;20(1):32. doi: 10.1186/s12942-021-00285-x [published Online First: 2021/07/04]

10. Wei Lam SS, Zhang ZC, Oh HC, et al. Reducing ambulance response times using discrete event simulation. Prehospital emergency care : official journal of the National Association of EMS Physicians and the National Association of State EMS Directors 2014;18(2):207-16. doi: 10.3109/10903127.2013.836266 [published Online First: 2013/10/19]

11. Lam SS, Zhang J, Zhang ZC, et al. Dynamic ambulance reallocation for the reduction of ambulance response times using system status management. The American journal of emergency medicine 2015;33(2):159-66. doi: 10.1016/j.ajem.2014.10.044 [published Online First: 2014/12/10]

12. Lam SSW, Ng CBL, Nguyen F, et al. Simulation-based decision support framework for dynamic ambulance redeployment in Singapore. Int J Med Inform 2017;106:37-47. doi: 10.1016/j.ijmedinf.2017.06.005 [published Online First: 2017/09/06]

13. Nogueira LC, Jr., Pinto LR, Silva PM. Reducing Emergency Medical Service response time via the reallocation of ambulance bases. Health Care Manag Sci 2016;19(1):31-42. doi: 10.1007/s10729-014-9280-4 [published Online First: 2014/04/20]

14. Roa JC, Escobar, J.W., Marín- Moreno, C.A. An online real-time matheuristic algorithm for dispatch and relocation of ambulances. International Journal of Industrial Engineering Computations 2020;11(3):443-68.

15. Schmid V. Solving the dynamic ambulance relocation and dispatching problem using approximate dynamic programming. Eur J Oper Res 2012;219(3):611-21. doi: 10.1016/j.ejor.2011.10.043 [published Online First: 2012/06/16]

16. Strauss C, Bildstein G, Efe J, et al. Optimizing Emergency Medical Service Structures Using a Rule-Based Discrete Event Simulation-A Practitioner's Point of View. Int J Environ Res Public Health 2021;18(5) doi: 10.3390/ijerph18052649 [published Online First: 2021/04/04]

17. Swalehe M, Aktas SG. Dynamic Ambulance Deployment to Reduce Ambulance Response Times Using Geographic Information Systems: A Case Study of Odunpazari District of Eskisehir Province, Turkey. Procedia Environmental Sciences 2016;36:199-206. doi: https://doi.org/10.1016/j.proenv.2016.09.033

18. van Buuren M, Jagtenberg, C., van Barneveld, T.,van der Mei, R., Bhulai, S. Ambulance Dispatch Center Pilots Proactive Relocation Policies to Enhance Effectiveness. Interfaces 2018;48(3):235-46.

For peer review only

Preferred Reporting Items for Systematic reviews and Meta-Analyses extension for Scoping Reviews (PRISMA-ScR) Checklist

SECTION	ITEM	PRISMA-ScR CHECKLIST ITEM	REPORTED ON PAGE #
**TITLE**			
Title	1	Identify the report as a scoping review.	1
**ABSTRACT**			
Structured summary	2	Provide a structured summary that includes (as applicable): background, objectives, eligibility criteria, sources of evidence, charting methods, results, and conclusions that relate to the review questions and objectives.	2
**INTRODUCTION**			
Rationale	3	Describe the rationale for the review in the context of what is already known. Explain why the review questions/objectives lend themselves to a scoping review approach.	3
Objectives	4	Provide an explicit statement of the questions and objectives being addressed with reference to their key elements (e.g., population or participants, concepts, and context) or other relevant key elements used to conceptualize the review questions and/or objectives.	3
**METHODS**			
Protocol and registration	5	Indicate whether a review protocol exists; state if and where it can be accessed (e.g., a Web address); and if available, provide registration information, including the registration number.	N/A
Eligibility criteria	6	Specify characteristics of the sources of evidence used as eligibility criteria (e.g., years considered, language, and publication status), and provide a rationale.	4
Information sources*	7	Describe all information sources in the search (e.g., databases with dates of coverage and contact with authors to identify additional sources), as well as the date the most recent search was executed.	4
Search	8	Present the full electronic search strategy for at least 1 database, including any limits used, such that it could be repeated.	4
Selection of sources of evidence†	9	State the process for selecting sources of evidence (i.e., screening and eligibility) included in the scoping review.	4
Data charting process‡	10	Describe the methods of charting data from the included sources of evidence (e.g., calibrated forms or forms that have been tested by the team before their use, and whether data charting was done independently or in duplicate) and any processes for obtaining and confirming data from investigators.	4
Data items	11	List and define all variables for which data were sought and any assumptions and simplifications made.	4, Table 2
Critical appraisal of individual sources of evidence§	12	If done, provide a rationale for conducting a critical appraisal of included sources of evidence; describe the methods used and how this information was used in any data synthesis (if appropriate).	Table 2, Supple matrl
Synthesis of results	13	Describe the methods of handling and summarizing the data that were charted.	4

For peer review only - http://bmjopen.bmj.com/site/about/guidelines.xhtml

SECTION	ITEM	PRISMA-ScR CHECKLIST ITEM	REPORTED ON PAGE #
**RESULTS**			
Selection of sources of evidence	14	Give numbers of sources of evidence screened, assessed for eligibility, and included in the review, with reasons for exclusions at each stage, ideally using a flow diagram.	5
Characteristics of sources of evidence	15	For each source of evidence, present characteristics for which data were charted and provide the citations.	5-6
Critical appraisal within sources of evidence	16	If done, present data on critical appraisal of included sources of evidence (see item 12).	Supplementary
Results of individual sources of evidence	17	For each included source of evidence, present the relevant data that were charted that relate to the review questions and objectives.	Table 2
Synthesis of results	18	Summarize and/or present the charting results as they relate to the review questions and objectives.	5-6
**DISCUSSION**			
Summary of evidence	19	Summarize the main results (including an overview of concepts, themes, and types of evidence available), link to the review questions and objectives, and consider the relevance to key groups.	8
Limitations	20	Discuss the limitations of the scoping review process.	8
Conclusions	21	Provide a general interpretation of the results with respect to the review questions and objectives, as well as potential implications and/or next steps.	8
**FUNDING**			
Funding	22	Describe sources of funding for the included sources of evidence, as well as sources of funding for the scoping review. Describe the role of the funders of the scoping review.	8

JBI = Joanna Briggs Institute; PRISMA-ScR = Preferred Reporting Items for Systematic reviews and Meta-Analyses extension for Scoping Reviews.
* Where *sources of evidence* (see second footnote) are compiled from, such as bibliographic databases, social media platforms, and Web sites.
† A more inclusive/heterogeneous term used to account for the different types of evidence or data sources (e.g., quantitative and/or qualitative research, expert opinion, and policy documents) that may be eligible in a scoping review as opposed to only studies. This is not to be confused with *information sources* (see first footnote).
‡ The frameworks by Arksey and O'Malley (6) and Levac and colleagues (7) and the JBI guidance (4, 5) refer to the process of data extraction in a scoping review as data charting.
§ The process of systematically examining research evidence to assess its validity, results, and relevance before using it to inform a decision. This term is used for items 12 and 19 instead of "risk of bias" (which is more applicable to systematic reviews of interventions) to include and acknowledge the various sources of evidence that may be used in a scoping review (e.g., quantitative and/or qualitative research, expert opinion, and policy document).

From: Tricco AC, Lillie E, Zarin W, O'Brien KK, Colquhoun H, Levac D, et al. PRISMA Extension for Scoping Reviews (PRISMAScR): Checklist and Explanation. Ann Intern Med. 2018;169:467–473. doi: 10.7326/M18-0850.

For peer review only - http://bmjopen.bmj.com/site/about/guidelines.xhtml

BMJ Open

Dynamic ambulance relocation: a scoping review

Journal:	*BMJ Open*
Manuscript ID	bmjopen-2023-073394.R1
Article Type:	Original research
Date Submitted by the Author:	16-Aug-2023
Complete List of Authors:	Becker, Julia; Institute for Disaster and Emergency Management<br>Kurland, Lisa; Örebro University; Örebro University Hospital<br>Höglund, Erik; Örebro University; Örebro Country Council, Ambulance Department<br>Hugelius, Karin; Örebro University; Örebro Country Council, Ambulance Department
**Primary Subject Heading**:	Emergency medicine
Secondary Subject Heading:	Emergency medicine
Keywords:	ACCIDENT & EMERGENCY MEDICINE, Organisation of health services < HEALTH SERVICES ADMINISTRATION & MANAGEMENT, Systematic Review, HEALTH SERVICES ADMINISTRATION & MANAGEMENT

SCHOLARONE™
Manuscripts

I, the Submitting Author has the right to grant and does grant on behalf of all authors of the Work (as defined in the below author licence), an exclusive licence and/or a non-exclusive licence for contributions from authors who are: i) UK Crown employees; ii) where BMJ has agreed a CC-BY licence shall apply, and/or iii) in accordance with the terms applicable for US Federal Government officers or employees acting as part of their official duties; on a worldwide, perpetual, irrevocable, royalty-free basis to BMJ Publishing Group Ltd ("BMJ") its licensees and where the relevant Journal is co-owned by BMJ to the co-owners of the Journal, to publish the Work in this journal and any other BMJ products and to exploit all rights, as set out in our licence.

The Submitting Author accepts and understands that any supply made under these terms is made by BMJ to the Submitting Author unless you are acting as an employee on behalf of your employer or a postgraduate student of an affiliated institution which is paying any applicable article publishing charge ("APC") for Open Access articles. Where the Submitting Author wishes to make the Work available on an Open Access basis (and intends to pay the relevant APC), the terms of reuse of such Open Access shall be governed by a Creative Commons licence – details of these licences and which Creative Commons licence will apply to this Work are set out in our licence referred to above.

Other than as permitted in any relevant BMJ Author's Self Archiving Policies, I confirm this Work has not been accepted for publication elsewhere, is not being considered for publication elsewhere and does not duplicate material already published. I confirm all authors consent to publication of this Work and authorise the granting of this licence.

For peer review only

1

Dynamic ambulance relocation: a scoping review

Authors:

Julia Becker[1], Lisa Kurland [2,3,4] Erik Höglund [2,4], Karin Hugelius [2,4]

1. Institute for Disaster and Emergency Management, Berlin, Germany
2. Faculty of Medicine and Health, Örebro University, Örebro, Sweden
3. Emergency Department, Örebro University Hospital, Örebro, Sweden
4. Ambulance Department, Örebro Country Council, Örebro, Sweden

* Corresponding author

Corresponding author contacts:

Karin Hugelius
E-mail: karin.hugelius@oru.se
Phone: +46 70 219 59 35

Word count: 3086

For peer review only

ABSTRACT

Objectives: Dynamic ambulance relocation means that the operators at a dispatch centre place an ambulance in a temporary location, with the goal of optimising coverage and response times in future medical emergencies. The aim of this study was to scope the current research on dynamic ambulance relocation.

Design: A scoping review was conducted using a structured search in PubMed, Scopus and Web of Science. In total, 21 papers were included. PRISMA Extension for Scoping Reviews was followed.

Results: Most papers described research with experimental designs involving the use of mathematical models to calculate the optimal use and temporary relocations of ambulances. The models relied on several variables, including distances, locations of hospitals, demographic-geological data, estimation of new emergencies, emergency medical services (EMS) working hours and other data. Some studies used historic ambulance dispatching data to develop models. Only one study reported a prospective, real-time evaluation of the models and the development of technical systems. No study reported on either positive or negative patient outcomes or real-life chain effects from the dynamic relocation of ambulances.

Conclusions: Current knowledge on dynamic relocation of ambulances is dominated by mathematical and technical support data that have calculated optimal locations of ambulance services based on response times and not patient outcomes. By contrast, knowledge of how patient outcomes and the working environment are affected by dynamic ambulance dispatching is lacking. This review has highlighted several gaps in the scientific coverage of the topic. The primary concern is the lack of studies reporting on patient outcomes, and the limited knowledge regarding several key factors, including the optimal use of ambulances in rural areas, turnaround times, domino effects and aspects of working environment for EMS personnel. Therefore, addressing these knowledge gaps is important in future studies.

Strengths and limitations of this study

- A broad search strategy was performed in scientific databases and a free text search in Google Scholar, including papers with both medical and technical scientific focus.
- A quality appraisal of included papers was added to the original scoping review method.
- A limitation was that the systematic search was conducted in three databases and included a limited number of papers reporting on dynamic relocation of ambulances from non-experimental settings and rural areas.
- Another limitation was the exclusion of editorials, non-academic papers and publications other than those published in-English.

BACKGROUND

Emergency medical services (EMS) are an essential part of the emergency care system. An increasing number of emergency calls and EMS use, and the resultant influx into the emergency department, are driving the need for optimal use of existing resources [1]. For this reason, considerable research has been devoted to optimising the use of the EMS from a dispatching and logistical perspective [2].

Dispatching ambulances is a complex task, as dispatch decisions include both strategic and operational considerations, such as the number and location of fixed ambulance stations and how the available ambulances should be used to optimise ambulance coverage [2]. The responsiveness of

For peer review only

the EMS depends on both stable factors, such as the location of a hospital, and factors that are constantly changing, such as traffic situations or weather conditions [3]. Two main models have been identified for operational decision-making on how best to use available resources and which ambulance to assign to a specific emergency call. The first model is the static location model (i.e. the ambulance is dispatched to a specific/given ambulance station and it returns to its designated ambulance station after a completed mission). The second model is dynamic relocation [2,4], also termed dynamic allocation or dynamic ambulance deployment. Dynamic relocation of ambulance vehicles means that the operator at the dispatch centre directs the ambulance to a temporary location (i.e. a location other than the regular, static ambulance station) with the purpose of reducing the response time. In this review, the term dynamic ambulance relocation was used to describe this kind of flexible repositioning of available ambulance resources. The use of dynamic ambulance relocation in Sweden varies, from not used at all to over 20 relocation missions per 1000 inhabitants [5]. In the Örebro County Council (Sweden), the number of dynamic ambulance relocation missions increased from 2% in 2021 to over 5% in 2022 [6].

Many studies have focused on technical solutions or relied on artificial intelligence to support ambulance dispatch operators in allocating the ambulance to the 'best' location [7]. Most of the focus has been on time. Prehospital time is a crucial factor for the survival of critically ill or injured patients [8,9]. In particular, response time (e.g. the time from dispatch until the ambulance is at the patient's side) [10] and turnaround time (e.g. the time from being dispatched until the ambulance is ready for new assignments) seems to be critical [11]. However, little is still known about the dispatch model and prehospital time, and no consensus has been reached regarding the most effective model for ambulance dispatching. The increased burden on the EMS system and the considerable variation in the use of dynamic ambulance relocation have created a need to summarise the current scientific knowledge on dynamic ambulance relocation and to identify knowledge gaps in the field. Therefore, the aim of this review is to scope the current research on dynamic ambulance relocation.

METHODS

A scoping review was conducted using the Arksey & O'Malley methodology [12]. The PRISMA Extension for Scoping Reviews (PRISMA-ScR) protocol was followed and used to report the study.

Stage 1: Identifying the research question

The research question for this study was 'What is known about dynamic ambulance allocations?'

Stage 2: Identifying relevant studies (search strategy)

Structured searches were performed in the PubMed database, Scopus and Web of Science by one author (KH) and an academic librarian The MesH terms 'ambulance' or 'emergency medical service' were combined with the keywords 'dispatch', 'dynamic' and 'relocation' as well as the MesH term 'machine learning', using truncation and Boolean operators (see Supplementary Material 1). The database searches were conducted on October 27 and December 09, 2022 and repeated in July 17, 2023. The searches were limited to papers published in English between 2012 and 2022. Papers with early or online publication, searchable in 2022, were included even if the final date of publication was in 2023. In addition, a manual search in Google Scholar, using free text search with the terms 'dynamic ambulance' and 'ambulance relocation', sorted by 'best match' was performed in December 2022 by the third author (EH), which retrieved two additional papers, and repeated in July 2023 rending additional one paper.

Stage 3: Study selection

The study selection process was conducted using Covidence software (Covidence systematic review software, Veritas Health Innovation, Melbourne, Australia). All papers identified in the literature

For peer review only

search (n=479) were exported to the Covidence systematic review software, where duplications were sought and removed. Thereafter, all records were screened by the first and second authors (JB and KH) by reading the title and abstract. For inclusion, an original research paper had to report on dynamic ambulance allocation for road ambulances and be published between 2012 and 2022. Both qualitative and quantitative studies were included. Papers reporting on air ambulance services, fixed locations of ambulance resources (such as the optimal location of an ambulance station), review studies, case studies or editorial texts were excluded. The full-text review assessed the entire paper and its eligibility for the study. In this stage of the selection process, the reason for exclusion was documented (see Figure 1). One disagreement occurred during the selection process, and it was solved by involving the second author (LK) in the decision.

In addition to the original scoping review methodology, a quality appraisal was performed [13], using the Critical Appraisal Skills Programme (CASPS) checklist for cohort studies [14]. The quality appraisal was performed by the first author (JB) and validated by the fourth author (KH) (see Supplementary Material 2).

Stage 4: Charting the data

All included studies were reviewed, and the following information was extracted: author(s) and year of publication, study location, design, aim, focus/ aim of the study, setting (urban and/or rural areas), type of modelling, variables used, main findings and quality appraisal.

Stage 5: Collating, summarising and reporting the results.

The results were synthesised and reported according to three domains; (1) study contexts and designs, (2) outcome variables and variables used in predictions of optimal ambulance dispatching and/ or relocation and (3) outcomes from dynamic ambulance relocation.

Patient and Public Involvement statement

No patients were included in the study.

Figure 1: Overview of the selection process and reasons for exclusion.

RESULTS

In total, 21 original papers were included in this scoping review. The included articles were published between 2012 and 2022, and the studies were conducted in Austria (n=1), Brazil (n=1), Canada (n=1), China (n=1), Colombia (n=1), Germany (n=1), Indonesia (n=1), Iran (n=2), Portugal (n=1), Singapore (n=3), Slovakia (n=1), Spain (n=1), Switzerland (n=1), the Netherlands (n=3), Turkey (n=1) and the USA (n=1). The studies were assessed as medium (n=16) or high (n=5) in quality (see Supplementary Material 2).

Study contexts and designs

The studies were most often conducted in urban areas, including metropolitan cities. Few studies were conducted in rural areas (see Supplementary Material 3). One study [15] was only based on emergency calls for critical patients.

All studies were based on mathematical simulation models, with a large variation of outcome variables. Most studies (n=12) used an experimental design and simulations. Of these, most (n=8)

For peer review only

simulations were based on retrospective EMS data and medical emergencies within a specified region (see Table 1 and Supplementary Material 3). Seven studies used experimental design (theoretical applications with no simulation) and two studies reported a prospective, real-time evaluation of the models and technical systems (see Supplementary Material 3). Two studies evaluated their developed models in real-time evaluations, one in both urban and rural areas [16] and one in an urban context [17].

Outcome variables and variables used in predictions of optimal ambulance dispatching and/ or relocation

Most studies (n=17) used response time as the primary outcome variable for the models developed and/ or tested. The information used to develop the mathematical models and/or computer-based programs was collected from retrospective data from real EMS activities (n=6), geographic and demographic data from national databases or Google Earth (n=6), estimations made by the research teams (n=3) or a combination of these sources within the same study (n=3) (see Supplementary Material 3). Some studies did not specify the sources for their variable data (n=10). All studies included three or more variables in their models.

Table 1. Summary of included studies

		Number of studies* N= 21
**Study design**	Experimental design and simulations	12
	Experimental design with no simulation/ testing	7
	Experimental design with real-time evaluation of models	2
**Data used**	Retrospective data from EMS activities	4
	Geographic and demographic data from national databases or Google Earth	8
	Estimations made by the research teams	3
	Combinations of estimations and official data	3
	No information given	3
**Study context**	Urban areas	12
	Rural areas	2
	Both urban and rural areas	4
	No information given	2
**Outcome variable**	Response time	17
	Turnaround time	3
	Workload on each ambulance	2
	Availability for new calls	2
	Perceived feasibility of software	1
	Travel distance per ambulance	1
**Variables used in the models**	Travel times and road network	14
	Current location of ambulances	13
	Number of available ambulances	9
	Location of hospitals	6
	Population living in a defined geographical area	6
	Time of day	4
	Response time	7
	Expected occurrence of medical emergencies within a specified area or timeframe	5
	Type of emergency or priority of the call	3
	Turnaround time	2

For peer review only

6

	Time at scene	2
	Uncovered emergencies	1
	Economical costs	1
	EMS working hours	4
	EMS workload	2
	Chain relocation effects	1

*Each study could include several variables

Outcomes from dynamic ambulance relocation

No study reported or discussed medical outcomes or clinical effects of dynamic relocation of ambulances. Four studies reported decreased response times when using dynamic relocation compared to ordinary dispatching and when using ordinary ambulance stations. Reported time saved was 130-160 seconds [18], 44 seconds [19], 54 seconds [20] and undefined saving of time [71]. In one study, the average percentage of calls reached within 9 min was improved with 43% up to 55% in different simulations [18]. However, the same study showed that an increase in total travelled distance by 7.9% to 31.7% [18].

No study reported on changes in response time or turnaround time in rural areas. Chain effects on other emergency calls due to dynamic ambulance relocations were reported [16]. One study reported that dynamic ambulance relocation reduced the total working time of all ambulances by about 9% per shift [17].

DISCUSSION

This review showed that most studies on dynamic ambulance relocation were based on experimental study designs and presented mathematical models in which three or more variables were used to calculate dynamic relocation of an ambulance with respect to response time. Most of the variables used in the models were fixed, and few studies evaluated the models or programs in prospective, real-life settings. Most studies were conducted in rural areas. No study reported on patient outcomes attained using dynamic ambulance relocation.

Ambulance dispatching and the optimal location of ambulances are important but challenging questions to analyse. Most variables used in the models presented were static, gathered from GIS systems or similar techniques. New techniques, such as artificial intelligence (AI), may offer additional tools for decision-making and dispatching of ambulances in the future [21]. However, dispatchers also use other variables, such as medical priority, type of medical care required, clinical judgement and their own professional experience, when making decisions on ambulance dispatching [22]. Therefore, an important consideration is to identify how fixed variables can be incorporated into decision-making systems, while also recognising the limitations of these tools.

No consensus has yet been reached on how to assess, define or measure the quality of care within the prehospital setting [23]; however, prehospital response times have long been a core component in these discussions [10]. Moreover, response times are often available for research and are (therefore) used as a proxy for outcome, which is also reflected in the results as most of the studies included in this review used response time as the primary outcome. However, trauma has traditionally been used as a role model for time-critical medical conditions, as prolonged response time is associated with increased mortality in trauma victims [24], especially in rural areas [11] . Studies suggest that prolonged prehospital time may have positive, negative and neutral associations with trauma patient mortality [25]. Patients with critical conditions, such as cardiac arrests, are likely to benefit from also small improvements in response time. However, the actual time gained by using

For peer review only - http://bmjopen.bmj.com/site/about/guidelines.xhtml

dynamic ambulance relocations, in comparison to static ambulance allocation (e.g. the ordinary ambulance station), was only studied in three of the articles, and in those, the time saved rendered from a few seconds up to two minutes. Hence, whether this saved time had clinical relevance (i.e. affected the outcome of the patients) cannot be determined as it was not measured. Dynamic ambulance relocation strategies focusing solely on shortening the average response time might be counter-productive when trying to save lives and minimize harm, especially in rural areas, since it may case longer response times or turnaround times for other patients. The goal must be to reach as many patients as possible with time-critical conditions "fast enough", not shortening the average response- or turnaround times. Reaching some patients fast enough might mean that other patients must wait longer for an ambulance. Less populated areas might have to rely on other rapid response units for the initial and potentially life-saving response before an ambulance can arrive. Since prehospital time is most likely not the only variable effecting clinical outcomes and quality of service within the ambulance services, variables such as early identification of time critical conditions, prehospital medical interventions provided [24] and the actual way of driving the ambulance [26] needs to be taken into consideration when discussing the *optimal* ambulance service. Studies evaluating the effect of ambulance relocation with respect to patient outcome with respect to the global effect of the relocation, ie not only on singular medical conditions but rather the effect on the patient population serviced by the relocated ambulances in real life contexts are strongly needed.

This review also does not allow any judgement of the value of dynamic relocation based on turnaround times, as only two of the included studies reported turnaround times. Both of these studies were conducted in large cities with many available emergency departments and neither compared turnaround times when dispatching using fixed ambulance locations versus dynamic relocation. Dynamic ambulance relocations may also cause a chain reaction, as temporary relocation of one ambulance may lead to a lack of resources in the original location, thus leading to new relocations and so on. These effects were covered in only one of the included studies. Therefore, no conclusions can be drawn regarding reductions in the response- or turnaround time by dynamic ambulance relocation based on the published studies.

Most studies developed and evaluated models based on EMS in urban areas. Notable differences exist between urban and rural areas regarding ambulance coverage, and turnaround time is most likely shorter in a city than in rural areas. A shortage of ambulance resources may therefore be more critical in rural areas than in urban areas. If the number of ambulances is small, the effect of a shortage or temporary relocation of one ambulance is more noticeable than in a city with more ambulances per area [27]. Conversely, the statistical risk for the occurrence of a time-critical condition is considerably lower in rural areas with small populations than in cities with large populations. However, the distances might be longer in rural areas than in urban areas. Even a temporary relocation may cause a significant delay in EMS response time; therefore, the effects of dynamic relocation might be more prominent in rural areas. Given the vulnerability of EMS coverage in rural areas, dynamic ambulance relocation in these areas needs to be prioritised in future studies. An important point made by Bélanger et al [18] is that dynamic ambulance relocation strategies could not compensate for a general lack of resources in a longer perspective and had side effects such as prolonged travelled distance and related costs. This was also the only identified study reflecting on optimal ambulance use from an economical or environmental perspective.

The well-being and working environment of EMS personnel is another relevant aspect of the optimal use of available ambulances. Long working hours, shift work and short times between the missions used for recovery are associated with work-related stress and mental health problems [28]. A practical consequence of dynamic ambulance relocation can be limited access to a toilet, kitchen or

For peer review only

resting facilities for ambulance personnel. Hence, human resource management may be more challenging when using dynamic dispatch strategies compared to fixed ambulance locations [18]. In this review, only one model included variables associated with the ambulance personnel working times, shifts and night rest. Only one study reported on workload effects. Working hours, working environment and workload are all important for both the effectiveness and efficiency of the EMS and should be considered when creating models for dynamic ambulance relocations.

Limitations

This scoping review has several limitations. One was that the search was performed in three databases. However, these provided a broad selection of both medical and non-medical journals and were therefore considered sufficient. By including studies from medical, technical and mathematical disciplines, the format for paper presentations varied. According to the scoping review methodology, only data from the results section should be charted and used in the review results. However, since the structure of the included papers varied, valuable information or outcomes stated under other headings were also extracted and included in the results of this review if the data or outcome referred to the actual study. The inconsistent terminology and definitions used for ambulance dispatching and dynamic relocation may have narrowed the search. A quality appraisal is not mandatory when using the scoping review methodology. However, to judge the value of the findings, a quality appraisal was found to add value and was therefore conducted.

CONCLUSION

The current knowledge on dynamic relocation emanates from mathematical and technical support used to calculate the optimal locations of ambulance services. However, knowledge of how patient outcomes and working environments are affected by dynamic ambulance dispatching is lacking. This review has highlighted several gaps in the scientific coverage of the topic. The primary concerns are the lack of studies reporting on patient outcomes and the limited knowledge about several key factors, including the optimal use of ambulances in rural areas, turnaround times, chain effects and aspects of working environment for EMS personnel. Therefore, addressing these knowledge gaps will be important in future studies.

List of abbreviations

Emergency Medical Services (EMS)

PRISMA Extension for Scoping Reviews (PRISMA-ScR)

Funding

This research received no specific grant from any funding agency in the public, commercial or not-for-profit sectors.

DECLARATIONS

Ethics approval and consent to participate
This study does not involve participants; therefore, ethical approval or consent was not required.

Availability of data and materials
Data sharing was not applicable, as no data sets were generated and/or analysed in this study.

Competing interests
The authors declare that they have no competing interests.

For peer review only

9

Author contributions

JB: Conceptualization, data collection, analysis, writing of the manuscript LK: Conceptualization, analysis, writing of the manuscript, EH: Conceptualization, data collection, confirming analysis, writing of the manuscript, KH: Conceptualization, data collection, analysis, writing and editing of the manuscript and revision. All authors read and approved the final manuscript.

References

1. Andrew E, Nehme Z, Cameron P, *et al.* Drivers of increasing emergency ambulance demand. *Prehosp Emerg Care* 2020;24(3):385-93. doi: 10.1080/10903127.2019.1635670 [published Online First: 2019/06/27]
2. Bélanger V, Ruiz A, Soriano P. Recent optimization models and trends in location, relocation, and dispatching of emergency medical vehicles. *Eur J Oper Res* 2019;272(1):1-23. doi: 10.1016/j.ejor.2018.02.055
3. Jánošíková L, Jankovič P, Kvet M, *et al.* Reorganization of an emergency medical system in a mixed urban-rural area. *Int J Environ Res Public Health* 2022;19(19):12369. doi: 10.3390/ijerph191912369 [published Online First: 2022/10/15]
4. Schmid V, Doerner KF. Ambulance location and relocation problems with time-dependent travel times. *Eur J Oper Res* 2010;207(3):1293-303. doi: 10.1016/j.ejor.2010.06.033
5. Nysam. Ambulanssjukvård. Nyckeltal 2019. Rapport Maj 2020. (In English: Ambulance care. Statistics 2019. Report May 2020). Stockholm, 2020. Available at: https://nysam.com/images/Rapporter/Ambulanssjukvard_2019_Nysamrapport.pdf
6. Örebro County Council AD. Statistics. Örebro, Sweden, 2023.
7. Neira-Rodado D, Escobar-Velasquez JW, McClean S. Ambulances deployment problems: categorization, evolution and dynamic problems review. *ISPRS Int J Geo-Inf* 2022;11(2):109. doi: 10.3390/ijgi11020109
8. Chen X, Guyette FX, Peitzman AB, *et al.* Identifying patients with time-sensitive injuries: Association of mortality with increasing prehospital time. *J Trauma Acute Care Surg* 2019;86(6):1015-22. doi: 10.1097/ta.0000000000002251 [published Online First: 2019/05/28]
9. van Rein EAJ, van der Sluijs R, Houwert RM, *et al.* Effectiveness of prehospital trauma triage systems in selecting severely injured patients: Is comparative analysis possible? *Am J Emerg Med* 2018;36(6):1060-69. doi: 10.1016/j.ajem.2018.01.055 [published Online First: 2018/02/06]
10. El Sayed MJ. Measuring quality in emergency medical services: a review of clinical performance indicators. *Emerg Med Int* 2012;2012:161630. doi: 10.1155/2012/161630 [published Online First: 2011/11/03]
11. Harmsen AM, Giannakopoulos GF, Moerbeek PR, *et al.* The influence of prehospital time on trauma patients outcome: a systematic review. *Injury* 2015;46(4):602-9. doi: 10.1016/j.injury.2015.01.008 [published Online First: 2015/01/30]
12. Arksey H, O'Malley, L. Scoping studies: towards a methodological framework. *Int J Soc Res Methodol: Theory & Pract* 2005;8(1):19-32. doi: 10.1080/1364557032000119616
13. Sucharew H, Macaluso M. Progress notes: methods for research evidence synthesis: the scoping review approach. *J Hosp Med* 2019;14(7):416-18. doi: 10.12788/jhm.3248 [published Online First: 2019/06/30]
14. (CASP) CASP. CASP Coort Study Checklists Available at: https://casp-uk.net/casp-tools-checklists/: CASP; 2020 [accessed November 04 2020].
15. Billhardt H, Lujak M, Sánchez-Brunete V, *et al.* Dynamic coordination of ambulances for emergency medical assistance services. *Knowl Based Syst* 2014;70:268-80. doi: 10.1016/j.knosys.2014.07.006

For peer review only

10

16. van Buuren M, Jagtenberg, C., van Barneveld, T.,van der Mei, R., Bhulai, S. Ambulance dispatch center pilots proactive relocation policies to enhance effectiveness. *Interfaces* 2018;48(3):235-46. doi: 10.1287/inte.2017.0936
17. Hajiali M, Teimoury E, Rabiee M, *et al.* An interactive decision support system for real-time ambulance relocation with priority guidelines. *Decis Support Syst* 2022;155:113712. doi: 10.1016/j.dss.2021.113712
18. Bélanger V, Kergosien Y, Ruiz A, Soriano P. An empirical comparison of relocation strategies in real-time ambulance fleet management. *Comput. Ind. Eng*. 2016;94:216-29. doi.org:/10.1016/j.cie.2016.01.023
19. Lam SS, Zhang J, Zhang ZC, *et al.* Dynamic ambulance reallocation for the reduction of ambulance response times using system status management. *Am J Emerg Med* 2015;33(2):159-66. doi: 10.1016/j.ajem.2014.10.044 [published Online First: 2014/12/10]
20. Deng Y, Zhang Y, Pan J. Optimization for locating emergency medical service facilities: a case study for health planning from China. *Risk Manag Healthc Policy* 2021;14:1791-802. doi: 10.2147/rmhp.S304475 [published Online First: 2021/05/11]
21. Ceklic E, Ball S, Finn J, *et al.* Ambulance dispatch prioritisation for traffic crashes using machine learning: A natural language approach. *Int J Med Inform* 2022;168:104886. doi: 10.1016/j.ijmedinf.2022.104886 [published Online First: 2022/10/29]
22. Hosseini SMR, Maleki M, Gorji HA, *et al.* Factors affecting emergency medical dispatchers' decision-making: a qualitative study. *J Multidiscip Healthc* 2018;11:391-98. doi: 10.2147/jmdh.S159593 [published Online First: 2018/09/04]
23. Rehn M, Kruger AJ. Quality improvement in pre-hospital critical care: increased value through research and publication. *Scand J Trauma, Resusc Emerg Med* 2014;22:34. doi: 10.1186/1757-7241-22-34 [published Online First: 2014/06/03]
24. Brown JB, Rosengart MR, Forsythe RM, *et al*. Not all prehospital time is equal: Influence of scene time on mortality. *J Trauma Acute Care Surg* 2016;81(1):93-100. doi: 10.1097/ta.0000000000000999 [published Online First: 2016/02/18]
25. Bedard AF, Mata LV, Dymond C, *et al*. A scoping review of worldwide studies evaluating the effects of prehospital time on trauma outcomes. *Int J Emerg Med* 2020;13(1):64. doi: 10.1186/s12245-020-00324-7 [published Online First: 2020/12/11]
26. Becker J, Hugelius K. Driving the ambulance: an essential component of emergency medical services: an integrative review. *BMC emerg med*. 2021;21(1):160. doi: 10.1186/s12873-021-00554-9.
27. van Barneveld TC, Bhulai S, van der Mei RD. A dynamic ambulance management model for rural areas : Computing redeployment actions for relevant performance measures. *Health Care Manag Sci* 2017;20(2):165-86. doi: 10.1007/s10729-015-9341-3 [published Online First: 2015/10/05]
28. Lawn S, Roberts L, Willis E, *et al.* The effects of emergency medical service work on the psychological, physical, and social well-being of ambulance personnel: a systematic review of qualitative research. *BMC Psychiatry* 2020;20(1):348. doi: 10.1186/s12888-020-02752-4 [published Online First: 2020/07/06]

For peer review only

11

For peer review only

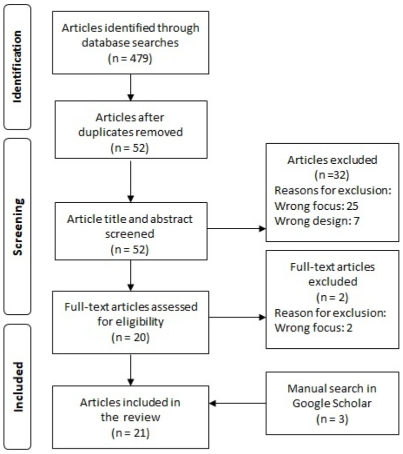

Figure 1Overview of selection process

304x171mm (96 x 96 DPI)

Supplementary material 1; Search strategy

Search strategy for each database

Pubmed (NLM). Search performed October 27, 2022, updated July, 17 2023: 75 abstracts

Ambulance, emergency medical services, dispatch, dynamic, dynamic allocation, relocation, machine learning		
	1.	Search: (((ambulance[MeSH Terms]) OR (emergency medical service[MeSH Terms])) AND ((dispatch*[Title/Abstract]) OR (allocation[Title/Abstract]))) AND ((((dynamic*[Title/Abstract]) OR (dynamic allocation*[Title/Abstract])) OR (relocation*[Title/Abstract])) OR (machine learning[MeSH Terms])) Filters: English, from 2012 - 2022
	2.	1 AND Filters: English, from 2012 - 2022

Web of Science Core Collection (Clarivate) (1975-present) Search performed October 27, 2022, updated July 17, 2023: 257 abstracts

Indexes=SCI-EXPANDED, SSCI, A&HCI, CPCI-S, CPCI-SSH, ESCI Timespan=All years

Ambulance, emergency medical service		
	1.	ALL=(((ambulance OR emergency medical service)))
Dispatch		
	2.	ALL=((dispatch*)) OR ((allocation*))
Dynamic, dynamic allocation, relocation, machine learning		
	3.	(((ALL=(dynamic*)) OR ALL=("dynamic allocation")) OR ALL=(relocation)) OR ALL=(machine learning)
Combined Sets		
	4.	1 AND 2 AND 3
Limits		
	6.	4 and English (Languages) and 2022 or 2021 or 2020 or 2019 or 2018 or 2017 or 2016 or 2015 or 2014 or 2013 or 2012 (Publication Years)

Scopus (Elsevier). Search performed December 09, 2022, updated July 17 2023: 147 abstracts

Ambulance, emergency medical services		
	1.	(TITLE-ABS-KEY (ambulance*) OR TITLE-ABS-KEY (emergency AND medical AND service*))

For peer review only

Dispatch		
	2.	TITLE-ABS-KEY (dispatch*)
Dynamic, relocation, machine learning		
	3.	(TITLE-ABS-KEY (dynamic*) OR TITLE-ABS-KEY (relocation*) OR TITLE-ABS-KEY (machine AND learning))
Combined Sets		
	4.	1 AND 2 AND 3<br>((TITLE-ABS-KEY (ambulance*) OR TITLE-ABS-KEY (emergency AND medical AND service*))) AND (TITLE-ABS-KEY (dispatch*)) AND ((TITLE-ABS-KEY (dynamic*) OR TITLE-ABS-KEY (relocation*) OR TITLE-ABS-KEY (machine AND learning)))
Limits		
	8.	7 AND((TITLE-ABS-KEY (ambulance*) OR TITLE-ABS-KEY (emergency AND medical AND service*))) AND (TITLE-ABS-KEY (dispatch*)) AND ((TITLE-ABS-KEY (dynamic*) OR TITLE-ABS-KEY (relocation*) OR TITLE-ABS-KEY (machine AND learning))) AND PUBYEAR > 2011 AND PUBYEAR < 2024

Google Scholar Search performed December, 2022, updated July 20 2023. Manual search.

Dynamic ambulance		
	1.	‘dynamic ambulance’
Ambulance relocation		
	2.	‘ambulance relocation’
Limits		
	3.	(Publication Years 2012-2022). Sorted: Best match.

For peer review only

Supplementary material 2: Quality appraisal according to CASP protocols

Author/ year	Did the study address a clearly focused issue?	Was the cohort requited in an acceptable way?	Was the exposure accurately measured to minimize bias?	Was the outcome accurately measured to minimize bias?	Have the authors identified all important confounding factors?	Have con-founding factors in the design and/or analysis been taken into consideration?	Was the follow-up of subjects complete and long enough?	How precise are the results?	Do you believe in the results?	Can the results be applied to the local population?	Do the results of this study fit with other available evidence?	Overall quality
Barneveldt 2017	Yes	N/A	N/A	Some	Some	Some	Not able to judge	Some	Some	Yes	Yes	Medium
Bélanger et al 2016	Yes	N/A	N/A	Yes	Some	Yes	Not able to judge	Some	Yes	Yes	Yes	Medium
Billhart et al 2014	Yes	N/A	N/A	Some	No	Some	Yes	Some	Yes	Yes	Yes	Medium
Carvallo et al 2020	Yes	N/A	N/A	Some	Some	Yes	Yes	Some	Yes	Yes	Yes	High
Degel et al 2015	Yes	N/A	N/A	Some	Some	Yes	Yes	Some	Yes	Yes	Yes	Medium
Deng et al 2021	Yes	N/A	N/A	Some	Some	Some	N/A	Some	Some	Unclear	Yes	Medium
Enayati et al 2018	Yes	N/A	N/A	Some	Some	Some	Yes	Some	Some	Yes	Yes	Medium
Hajiali et al 2022	Yes	N/A	N/A	Yes	Some	Yes	No	Good	Some	Yes	Some	High
Jagtenberg et al 2017	Yes	N/A	N/A	Some	Some	Yes	N/A	Some	Some	Unclear	Yes	Medium
Jánošíková et al 2021	Yes	N/A	N/A	Some	Some	Some	Not able to judge	Some	Some	Yes	Yes	Medium
Karpova et al 2022	Yes	N/A	N/A	Some	Some	Some	Not able to judge	Some	Some	Yes	Yes	Medium
Lam et al 2014	Yes	N/A	N/A	Yes	Yes	Some	Yes	Good	Yes	Yes	Yes	High
Lam et al 2015	Yes	N/A	N/A	Yes	Some	Some	Yes	Some	Yes	Yes	Yes	Medium
Lam et al 2017	Yes	N/A	N/A	Yes	Yes	Yes	Yes	Some	Yes	Yes	Yes	High
Nogueira et al 2016	Yes	N/A	N/A	Some	Some	Some	Some	Some	Some	Yes	Yes	Medium
Roa et al 2020	Yes	N/A	N/A	Some	Some	Some	Some	Some	Some	Yes	Yes	Medium
Schmid 2021	Yes	N/A	N/A	Some	Some	Some	Yes	Some	Some	Yes	Yes	Medium
Strauss et al 2021	Yes	N/A	N/A	Some	Some	Some	Unclear	Some	Yes	Yes	Yes	Medium

For peer review only

Swalehe & Aktas 2016	Yes	N/A	N/A	Some	Some	Some	Unclear	Some	Yes	Yes	Yes	Medium
Umam et al 2022	Yes	N/A	N/A	Some	Some	Some	Unclear	Some	Yes	Yes	Yes	Medium
Van Buuren et al 2018	Yes	N/A	N/A	Yes	Some	Yes	Yes	Good	Yes	Yes	Yes	High

For peer review only

Supplementary material 3. Overview of included studies

Authors and publication year	Study location	Design	Aim of the study	Setting	Type of modelling	Variables used in the model	Outcome variable	Main findings	Quality appraisal
Barneveldt et al 2017[1]	The Netherlands	Experimental	To develop a method to obtain good relocation strategies in real time	Rural areas	Mathematic models and scenario probabilities based on retrospective data	Demand locations<br>Time of the day<br>Level of priority<br>Total number of ambulances<br>Treatment time on scene	Response time	A one-step look-ahead heuristic model that relocated ambulances to minimize the response time was presented	Medium quality
Bélanger et al 2016[2]	Canada	Experimental with simulation	To study outcomes of different location and relocation strategies.	Urban areas	Mathematic models and scenario probabilities	Available ambulances<br>Travel times<br>Ambulance stations locations<br>Population density<br>Expected emergency calls<br>Working shift time	Response time<br>Response rate within 9 minutes<br>Travelled distance	Using a flexible relocation strategy provides a better service. However, when the system is congested, dynamic strategies cannot compensate for the lack of resources	Medium quality
Billhardt et al 2014[3]	Spain	Experimental	To present an event-driven system for dynamic ambulance assignment and redeployment	Urban areas	Mathematic models and scenario probabilities	Geographical location<br>Available ambulances<br>(The study only included critical patients)	Response time	Mathematic models can reduce the response times for EMS services. However, more variables need to be considered and included in such models	Medium quality

For peer review only

Carvalho et al 2020[4]	Portugal	Experimental with simulation	To develop models to solve ambulance dispatching and relocation problems	Urban areas	Mathematic models and scenario probabilities based on retrospective data (n=20 000)	Available ambulances<br>Travel times<br>Ambulance stations locations<br>Dynamic relocation spots<br>Waiting time<br>Maximum response times<br>Uncovered emergencies	Availability for new calls	A mixed-integrated programming model and an experimental model was presented. Both approaches used a time preparedness measure to evaluate the system's capability to handle new emergencies	High quality
Degel et al 2015[5]	Germany	Experimental with simulation	To support decision on location planning for ambulances	Rural areas	Mathematic models and scenario probabilities based on retrospective data	Travel time<br>Required coverage<br>Variation in fleet<br>Flexible locations	Availability for new calls	The inclusion of time-dependent and empirical data had a substantial impact on the solution	Medium quality
Deng et al 2021[6]	China	Experimental	To optimize the EMS system	No information given	Mathematic models	Population density<br>Road network<br>Location of hospitals<br>Location of flexible locations	Response time	The solution presented could effectively achieve a population coverage within 15 minutes, with a minimum number of temporary ambulance points in addition to existing	Medium quality

For peer review only

								ambulance stations	
Enayati et al 2018[7]	USA	Experimental with simulation	To develop a real-time model to maximize coverage with minimum possible total travel time	Urban	Mathematic models and scenario probabilities based on retrospective data (n=50 000)	Initial fleet size of ambulances<br>Travel limit (max 20 min)<br>Maximum allowed workload of each ambulance<br>Day and time<br>Shift times (12h)	Turnaround time	The modeling of the real time deployment model resulted in improvement in average coverage and prevented accumulated workload for ambulance personnel	Medium quality
Hajiali et al, 2022[8]	Iran	Experimental real-life testing	To develop a decision support system for optimal ambulance coverage	Urban	Mathematic models and real-life evaluation (1 week) of response time and workload for each ambulance	Base location<br>Demand zones and time ranges from these (7 min, 15 min, 25 min)<br>Number of available ambulances<br>Relocation time<br>Working shift time (12h)	Response time<br>Workload	The decision support system decreased response times and reduced the total working time of all ambulances by about 9% per shift.	Hight quality
Jagtenberg et al 2017[9]	The Netherlands	Experimental with simulation	To present different dispatch rules and suggest a heuristic method for ambulance dispatching	Urban areas	Mathematic models and scenario probabilities	Ambulance station locations<br>Demand locations<br>Hospital locations<br>the base location<br>Time of the day<br>Driving time	Response time	Two methods to obtain ambulance dispatch policies were reported. Their effects could not be defined	Medium quality
Jánošíková et al 2021[10]	Slovakia	Experimental with simulation	To identify optimization	Urban and rural areas	Mathematic models and	Demand zones<br>Population (and age profile)	Response time	The EMS systems should be designed to	Medium quality

For peer review only

			criterion of the EMS		scenario probabilities	Temporary locations Travel time		minimize response times, and not to maximize the number of calls served within a given time limit	
Karpova et al 2022 (2023)[11]	Spain	Experimental	To addresses the problem of dynamic relocation of ambulances through the design and development of heuristic tools	Urban and rural areas	Mathematic models and scenario probabilities	Population coverage in demand zones Ordinary base location Driving time Possible relocation bases	Response time	The designed relocation algorithms perform better than if there was no relocation strategy.	Medium quality
Lam et al 2014[12]	Singapore	Experimental with simulation	To develop a discrete-event simulation model for the Singapore EMS	Urban area	Mathematic models and scenario probabilities based on retrospective data (n=54 548)	Geographical information systems (GIS) data, Travel time Ambulance location	Response time	Response times were improved via a more effective reallocation of ambulances and dispatch policy, without additional ambulances	High quality
Lam et al 2015[13]	Singapore	Experimental with simulation	To develop efficient ambulance allocation plans in Singapore and to evaluate its	Urban area	Mathematic models and scenario probabilities based on retrospective data (n= 52 512)	Call times Dispatch times, Scene arrival times, Hospital conveyance times Turnaround time, Locations of incident	Response time	Geographical information system-based plans reduced the median response times with 13 to 44 seconds	Medium

For peer review only

			performance using the DES model.					compared to a static reallocation policy	
Lam et al 2017[14]	Singapore	Experimental with simulation	To report on the previously reported work by leveraging on the DES model	Urban area	Mathematic models and scenario probabilities based on retrospective data (n=217 000)	Geographical coverage<br>Response Times<br>Turnaround times and utilization rates	Turnaround time	The integration of dynamic redeployments optimized the ambulance coverage	High quality
Nogueira et al 2016[15]	Brazil	Experimental with simulation	To analyze the EMS using two modeling techniques: optimization and simulation	Urban areas	Mathematic models and scenario probabilities	Ambulance station location<br>Demand posts<br>Hospital locations<br>Cost for each ambulance	Response time	A balanced number of ambulances allocated across certain bases at certain times improved the system performance without increasing the total number of ambulances	Medium quality
Roa et al 2020[16]	Colombia	Experimental with simulation	To design an algorithm to support the real-time operation of ambulances	Urban areas	Mathematic models and scenario probabilities based on retrospective data	Types of emergency,<br>Types of ambulances<br>Demand points<br>Locations for ambulances<br>Modelled on-site attention time<br>Travel times	Response time<br>Availability for new calls	The real-time management of ambulance fleets involved challenging decisions due to frequent, complex, and random changes in system conditions	Medium quality

For peer review only

Schmid 2021[17]	Austria	Experimental with simulation	To propose a stochastic dynamic model for the ambulance relocation and dispatching problem	Urban area	Mathematic models and scenario probabilities based on retrospective data (n=3748)	Location of hospitals and patients, response times, travel times, using data.	Response time	By deviating from the traditional rule of dispatching the closest ambulance available and relocating them to their home base after having finished serving, the coverage can be improved	Medium quality
Strauss et al 2021[18]	Switzerland and Germany	Experimental with simulation	To explain the major features of "rule-based discrete event simulation"	Urban and rural areas	Mathematic models and scenario probabilities	Response times, demands per day, travel time, dispatch priority	Response time	Rule-based DES models can improve regional emergency services' efficiency without increasing cost. All simulation-based methods suggest normative solutions and optimize EMS' performance within given healthcare system structures	Medium quality
Swalehe & Aktas 2016[19]	Turkey	Experimental	To find optimal ambulance	Urban	Computer based systems (one with	Time of day Population Driving time	Response time	Ambulance response time can be reduced	Medium quality

For peer review only

			locations (stations and temporary demand points)		information on retrospective emergency calls	Road network data		with a dynamic relocation plan taking time of the day in consideration. Dynamic ambulance deployment is a more effective ambulance deployment strategy than static ambulance deployment	
Umam et al 2022[20]	Indonesia	Experimental	To overcome the location problem and allocation of ambulances by using a combination of metaheuristics and simulation	No information given	Mathematic models	Ambulance station location<br>Demand posts<br>Travel times<br>Number of available ambulances	Response time	Involving several potential bases can produce a short response time.	Medium quality
Van Buuren et al 2018[21]	The Netherlands	Experimental real-life testing	To evaluate two dynamic relocation policies for operational use and implemented in a software tool for realtime	Urban and rural areas	Mathematic models and real-life evaluation (qual and quant)	Geographical distances<br>Population<br>Probability of new medical emergencies<br>Ambulance stations<br>Travel times<br>Chain relocation effects EMS sleepingtimes	Response time<br>Feasibility of the software	When dispatchers worked in a consistent way, relocation decisions were made faster, dispatchers had a better overview of the	High quality

For peer review only

	decision support	EMS shiftworking times	available ambulances. The use of policies instead of dispatcher intuition improved efficiency.

References for included studies

1. van Barneveld TC, Bhulai S, van der Mei RD. A dynamic ambulance management model for rural areas : Computing redeployment actions for relevant performance measures. Health Care Manag Sci 2017;20(2):165-86. doi: 10.1007/s10729-015-9341-3 [published Online First: 2015/10/05]

2. Bélanger V, Kergosien Y, Ruiz A, Soriano P. An empirical comparison of relocation strategies in real-time ambulance fleet management. Comput Ind Eng. 2016;94:216-29.

3. Billhardt H, Lujak M, Sánchez-Brunete V, et al. Dynamic coordination of ambulances for emergency medical assistance services. Knowledge-Based Systems 2014;70:268-80. doi: https://doi.org/10.1016/j.knosys.2014.07.006

4. Carvalho AS, Captivo ME, Marques I. Integrating the ambulance dispatching and relocation problems to maximize system's preparedness. European Journal of Operational Research 2020;283(3):1064-80. doi: https://doi.org/10.1016/j.ejor.2019.11.056

5. Degel D, Wiesche L, Rachuba S, et al. Time-dependent ambulance allocation considering data-driven empirically required coverage. Health Care Manag Sci 2015;18(4):444-58. doi: 10.1007/s10729-014-9271-5 [published Online First: 2014/03/13]

6. Deng Y, Zhang Y, Pan J. Optimization for Locating Emergency Medical Service Facilities: A Case Study for Health Planning from China. Risk Manag Healthc Policy 2021;14:1791-802. doi: 10.2147/rmhp.S304475 [published Online First: 2021/05/11]

7. Enayati S, Mayorga ME, Rajagopalan HK, et al. Real-time ambulance redeployment approach to improve service coverage with fair and restricted workload for EMS providers. Omega 2018;79:67-80. doi: https://doi.org/10.1016/j.omega.2017.08.001

8. Hajiali M, Teimoury E, Rabiee M, et al. An interactive decision support system for real-time ambulance relocation with priority guidelines. Decision Support Systems 2022;155:113712. doi: https://doi.org/10.1016/j.dss.2021.113712

9. Jagtenberg CJ, Bhulai S, van der Mei RD. Dynamic ambulance dispatching: is the closest-idle policy always optimal? Health Care Manag Sci 2017;20(4):517-31. doi: 10.1007/s10729-016-9368-0 [published Online First: 2016/05/22]

For peer review only

10. Jánošíková Ľ, Jankovič P, Kvet M, et al. Coverage versus response time objectives in ambulance location. Int J Health Geogr 2021;20(1):32. doi: 10.1186/s12942-021-00285-x [published Online First: 2021/07/04]

11. Karpova Y, Villa F, Vallada E, Vecina MÁ. Heuristic algorithms based on the isochron analysis for dynamic relocation of medical emergency vehicles. Expert Syst. Appl. 2023;212:118773. Available online Sept 2022.

12. Wei Lam SS, Zhang ZC, Oh HC, et al. Reducing ambulance response times using discrete event simulation. Prehospital emergency care : official journal of the National Association of EMS Physicians and the National Association of State EMS Directors 2014;18(2):207-16. doi: 10.3109/10903127.2013.836266 [published Online First: 2013/10/19]

13. Lam SS, Zhang J, Zhang ZC, et al. Dynamic ambulance reallocation for the reduction of ambulance response times using system status management. The American journal of emergency medicine 2015;33(2):159-66. doi: 10.1016/j.ajem.2014.10.044 [published Online First: 2014/12/10]

14. Lam SSW, Ng CBL, Nguyen F, et al. Simulation-based decision support framework for dynamic ambulance redeployment in Singapore. Int J Med Inform 2017;106:37-47. doi: 10.1016/j.ijmedinf.2017.06.005 [published Online First: 2017/09/06]

15. Nogueira LC, Jr., Pinto LR, Silva PM. Reducing Emergency Medical Service response time via the reallocation of ambulance bases. Health Care Manag Sci 2016;19(1):31-42. doi: 10.1007/s10729-014-9280-4 [published Online First: 2014/04/20]

16. Roa JC, Escobar, J.W., Marín- Moreno, C.A. An online real-time matheuristic algorithm for dispatch and relocation of ambulances. International Journal of Industrial Engineering Computations 2020;11(3):443-68.

17. Schmid V. Solving the dynamic ambulance relocation and dispatching problem using approximate dynamic programming. Eur J Oper Res 2012;219(3):611-21. doi: 10.1016/j.ejor.2011.10.043 [published Online First: 2012/06/16]

18. Strauss C, Bildstein G, Efe J, et al. Optimizing Emergency Medical Service Structures Using a Rule-Based Discrete Event Simulation-A Practitioner's Point of View. Int J Environ Res Public Health 2021;18(5) doi: 10.3390/ijerph18052649 [published Online First: 2021/04/04]

19. Swalehe M, Aktas SG. Dynamic Ambulance Deployment to Reduce Ambulance Response Times Using Geographic Information Systems: A Case Study of Odunpazari District of Eskisehir Province, Turkey. Procedia Environmental Sciences 2016;36:199-206. doi: https://doi.org/10.1016/j.proenv.2016.09.033

20. Umam, M. I. H., Santosa, B., & Siswanto, N. (2022). A Simulation Optimization for Location and Allocation of Emergency Medical Service. International Int. j. online biomed. eng, 18(11), pp. 158–172.

21. van Buuren M, Jagtenberg, C., van Barneveld, T.,van der Mei, R., Bhulai, S. Ambulance Dispatch Center Pilots Proactive Relocation Policies to Enhance Effectiveness. Interfaces 2018;48(3):235-46.

For peer review only

For peer review only

Preferred Reporting Items for Systematic reviews and Meta-Analyses extension for Scoping Reviews (PRISMA-ScR) Checklist

SECTION	ITEM	PRISMA-ScR CHECKLIST ITEM	REPORTED ON PAGE #
**TITLE**			
Title	1	Identify the report as a scoping review.	1
**ABSTRACT**			
Structured summary	2	Provide a structured summary that includes (as applicable): background, objectives, eligibility criteria, sources of evidence, charting methods, results, and conclusions that relate to the review questions and objectives.	2
**INTRODUCTION**			
Rationale	3	Describe the rationale for the review in the context of what is already known. Explain why the review questions/objectives lend themselves to a scoping review approach.	3
Objectives	4	Provide an explicit statement of the questions and objectives being addressed with reference to their key elements (e.g., population or participants, concepts, and context) or other relevant key elements used to conceptualize the review questions and/or objectives.	3
**METHODS**			
Protocol and registration	5	Indicate whether a review protocol exists; state if and where it can be accessed (e.g., a Web address); and if available, provide registration information, including the registration number.	N/A
Eligibility criteria	6	Specify characteristics of the sources of evidence used as eligibility criteria (e.g., years considered, language, and publication status), and provide a rationale.	4
Information sources*	7	Describe all information sources in the search (e.g., databases with dates of coverage and contact with authors to identify additional sources), as well as the date the most recent search was executed.	4
Search	8	Present the full electronic search strategy for at least 1 database, including any limits used, such that it could be repeated.	4
Selection of sources of evidence†	9	State the process for selecting sources of evidence (i.e., screening and eligibility) included in the scoping review.	4
Data charting process‡	10	Describe the methods of charting data from the included sources of evidence (e.g., calibrated forms or forms that have been tested by the team before their use, and whether data charting was done independently or in duplicate) and any processes for obtaining and confirming data from investigators.	4
Data items	11	List and define all variables for which data were sought and any assumptions and simplifications made.	4, Table 2
Critical appraisal of individual sources of evidence§	12	If done, provide a rationale for conducting a critical appraisal of included sources of evidence; describe the methods used and how this information was used in any data synthesis (if appropriate).	Table 2, Supple matrl
Synthesis of results	13	Describe the methods of handling and summarizing the data that were charted.	4

St. Michael's
Inspired Care.
Inspiring Science.

For peer review only - http://bmjopen.bmj.com/site/about/guidelines.xhtml

SECTION	ITEM	PRISMA-ScR CHECKLIST ITEM	REPORTED ON PAGE #
**RESULTS**			
Selection of sources of evidence	14	Give numbers of sources of evidence screened, assessed for eligibility, and included in the review, with reasons for exclusions at each stage, ideally using a flow diagram.	5
Characteristics of sources of evidence	15	For each source of evidence, present characteristics for which data were charted and provide the citations.	5-6
Critical appraisal within sources of evidence	16	If done, present data on critical appraisal of included sources of evidence (see item 12).	Supplementary
Results of individual sources of evidence	17	For each included source of evidence, present the relevant data that were charted that relate to the review questions and objectives.	Table 2
Synthesis of results	18	Summarize and/or present the charting results as they relate to the review questions and objectives.	5-6
**DISCUSSION**			
Summary of evidence	19	Summarize the main results (including an overview of concepts, themes, and types of evidence available), link to the review questions and objectives, and consider the relevance to key groups.	8
Limitations	20	Discuss the limitations of the scoping review process.	8
Conclusions	21	Provide a general interpretation of the results with respect to the review questions and objectives, as well as potential implications and/or next steps.	8
**FUNDING**			
Funding	22	Describe sources of funding for the included sources of evidence, as well as sources of funding for the scoping review. Describe the role of the funders of the scoping review.	8

JBI = Joanna Briggs Institute; PRISMA-ScR = Preferred Reporting Items for Systematic reviews and Meta-Analyses extension for Scoping Reviews.
* Where *sources of evidence* (see second footnote) are compiled from, such as bibliographic databases, social media platforms, and Web sites.
† A more inclusive/heterogeneous term used to account for the different types of evidence or data sources (e.g., quantitative and/or qualitative research, expert opinion, and policy documents) that may be eligible in a scoping review as opposed to only studies. This is not to be confused with *information sources* (see first footnote).
‡ The frameworks by Arksey and O'Malley (6) and Levac and colleagues (7) and the JBI guidance (4, 5) refer to the process of data extraction in a scoping review as data charting.
§ The process of systematically examining research evidence to assess its validity, results, and relevance before using it to inform a decision. This term is used for items 12 and 19 instead of "risk of bias" (which is more applicable to systematic reviews of interventions) to include and acknowledge the various sources of evidence that may be used in a scoping review (e.g., quantitative and/or qualitative research, expert opinion, and policy document).

From: Tricco AC, Lillie E, Zarin W, O'Brien KK, Colquhoun H, Levac D, et al. PRISMA Extension for Scoping Reviews (PRISMAScR): Checklist and Explanation. Ann Intern Med. 2018;169:467–473. doi: 10.7326/M18-0850.

For peer review only

BMJ Open

Dynamic ambulance relocation: a scoping review

Journal:	*BMJ Open*
Manuscript ID	bmjopen-2023-073394.R2
Article Type:	Original research
Date Submitted by the Author:	30-Oct-2023
Complete List of Authors:	Becker, Julia; Institute for Disaster and Emergency Management<br>Kurland, Lisa; Örebro University; Örebro University Hospital<br>Höglund, Erik; Örebro University; Örebro Country Council, Ambulance Department<br>Hugelius, Karin; Örebro University; Örebro Country Council, Ambulance Department
**Primary Subject Heading**:	Emergency medicine
Secondary Subject Heading:	Emergency medicine
Keywords:	ACCIDENT & EMERGENCY MEDICINE, Organisation of health services < HEALTH SERVICES ADMINISTRATION & MANAGEMENT, Systematic Review, HEALTH SERVICES ADMINISTRATION & MANAGEMENT

SCHOLARONE™
Manuscripts

I, the Submitting Author has the right to grant and does grant on behalf of all authors of the Work (as defined in the below author licence), an exclusive licence and/or a non-exclusive licence for contributions from authors who are: i) UK Crown employees; ii) where BMJ has agreed a CC-BY licence shall apply, and/or iii) in accordance with the terms applicable for US Federal Government officers or employees acting as part of their official duties; on a worldwide, perpetual, irrevocable, royalty-free basis to BMJ Publishing Group Ltd ("BMJ") its licensees and where the relevant Journal is co-owned by BMJ to the co-owners of the Journal, to publish the Work in this journal and any other BMJ products and to exploit all rights, as set out in our licence.

The Submitting Author accepts and understands that any supply made under these terms is made by BMJ to the Submitting Author unless you are acting as an employee on behalf of your employer or a postgraduate student of an affiliated institution which is paying any applicable article publishing charge ("APC") for Open Access articles. Where the Submitting Author wishes to make the Work available on an Open Access basis (and intends to pay the relevant APC), the terms of reuse of such Open Access shall be governed by a Creative Commons licence – details of these licences and which Creative Commons licence will apply to this Work are set out in our licence referred to above.

Other than as permitted in any relevant BMJ Author's Self Archiving Policies, I confirm this Work has not been accepted for publication elsewhere, is not being considered for publication elsewhere and does not duplicate material already published. I confirm all authors consent to publication of this Work and authorise the granting of this licence.

For peer review only

1

Dynamic ambulance relocation: a scoping review

Authors:

Julia Becker[1], Lisa Kurland [2,3,4] Erik Höglund [2,4], Karin Hugelius [2,4]

1. Institute for Disaster and Emergency Management, Berlin, Germany
2. Faculty of Medicine and Health, Örebro University, Örebro, Sweden
3. Emergency Department, Örebro University Hospital, Örebro, Sweden
4. Ambulance Department, Örebro Country Council, Örebro, Sweden

* Corresponding author

Corresponding author contacts:

Karin Hugelius
E-mail: karin.hugelius@oru.se
Phone: +46 70 219 59 35

Word count: 3016

For peer review only

ABSTRACT

Objectives**:** Dynamic ambulance relocation means that the operators at a dispatch centre place an ambulance in a temporary location, with the goal of optimising coverage and response times in future medical emergencies. The aim of this study was to scope the current research on dynamic ambulance relocation.

Design: A scoping review was conducted using a structured search in PubMed, Scopus and Web of Science. In total, 21 papers were included. PRISMA Extension for Scoping Reviews was followed.

Results: Most papers described research with experimental designs involving the use of mathematical models to calculate the optimal use and temporary relocations of ambulances. The models relied on several variables, including distances, locations of hospitals, demographic-geological data, estimation of new emergencies, emergency medical services (EMS) working hours and other data. Some studies used historic ambulance dispatching data to develop models. Only one study reported a prospective, real-time evaluation of the models and the development of technical systems. No study reported on either positive or negative patient outcomes or real-life chain effects from the dynamic relocation of ambulances.

Conclusions: Current knowledge on dynamic relocation of ambulances is dominated by mathematical and technical support data that have calculated optimal locations of ambulance services based on response times and not patient outcomes. Conversly, knowledge of how patient outcomes and the working environment are affected by dynamic ambulance dispatching is lacking. This review has highlighted several gaps in the scientific coverage of the topic. The primary concern is the lack of studies reporting on patient outcomes, and the limited knowledge regarding several key factors, including the optimal use of ambulances in rural areas, turnaround times, domino effects and aspects of working environment for EMS personnel. Therefore, addressing these knowledge gaps is important in future studies.

Strengths and limitations of this study

- A broad, structured search strategy including several pilot searches, was performed in scientific databases and included papers with both medical and technical focus.
- A quality appraisal of included papers was added to the original scoping review method.
- A limitation was that the systematic search was conducted in three databases and included a limited number of papers reporting on dynamic relocation of ambulances from non-experimental settings and rural areas.
- Another limitation was the exclusion of editorials, non-academic papers and publications other than those published in-English.

BACKGROUND

Emergency medical services (EMS) are an essential part of the emergency care system. An increasing number of emergency calls and EMS use, and the resultant influx into the emergency department, are driving the need for optimal use of existing resources [1]. For this reason, considerable research has been devoted to optimising the use of the EMS from a dispatching and logistical perspective [2].

Dispatching ambulances is a complex task, as dispatch decisions include both strategic and operational considerations, such as the number and location of fixed ambulance stations and how the available ambulances should be used to optimise ambulance coverage [2]. The responsiveness of

For peer review only

the EMS depends on both stable factors, such as the location of a hospital, and factors that are constantly changing, such as traffic situations or weather conditions [3]. Two main models have been identified for operational decision-making on how best to use available resources and which ambulance to assign to a specific emergency call. The first model is the static location model (i.e. the ambulance is dispatched to a specific/given ambulance station and it returns to its designated ambulance station after a completed mission). The second model is dynamic relocation [2,4], also termed dynamic allocation or dynamic ambulance deployment. Dynamic relocation of ambulance vehicles means that the operator at the dispatch centre directs the ambulance to a temporary location (i.e. a location other than the regular, static ambulance station) with the purpose of reducing the response time. In this review, the term dynamic ambulance relocation was used to describe this kind of flexible repositioning of available ambulance resources. The use of dynamic ambulance relocation in Sweden varies, from not used at all to over 20 relocation missions per 1000 inhabitants [5]. In the Örebro County Council (Sweden), the number of dynamic ambulance relocation missions increased from 2% in 2021 to over 5% in 2022 [6].

Many studies have focused on technical solutions or relied on artificial intelligence to support ambulance dispatch operators in allocating the ambulance to the 'best' location [7]. Most of the focus has been on time. Prehospital time is a crucial factor for the survival of critically ill or injured patients [8,9]. In particular, response time (e.g. the time from dispatch until the ambulance is at the patient's side) [10] and turnaround time (e.g. the time from being dispatched until the ambulance is ready for new assignments) seems to be critical [11]. However, little is still known about the dispatch model and prehospital time, and no consensus has been reached regarding the most effective model for ambulance dispatching. The increased burden on the EMS system and the considerable variation in the use of dynamic ambulance relocation have created a need to summarise the current scientific knowledge on dynamic ambulance relocation and to identify knowledge gaps in the field. Therefore, the aim of this review is to scope the current research on dynamic ambulance relocation.

METHODS

A scoping review was conducted using the Arksey & O'Malley methodology [12]. The PRISMA Extension for Scoping Reviews (PRISMA-ScR) protocol was followed and used to report the study.

Stage 1: Identifying the research question

The research question for this study was 'What is known about dynamic ambulance allocations?'

Stage 2: Identifying relevant studies (search strategy)

Structured searches were performed in the PubMed database, Scopus and Web of Science by one author (KH) and two academic librarians using bothMeSH terms and keywords , as well as truncation and Boolean operators (see Supplementary Material 1). Pilot searches were made in December 2022 and repeated in July 2023. The final search was made in October 2023. The searches were limited to papers published in English between 2012 and October 23, 2023. In addition, a manual search in Google Scholar, using free text search with the terms 'dynamic ambulance' and 'ambulance relocation', sorted by 'best match' was performed in September and October 2023 by two authors (EH and JB) individually, which retrieved three additional papers in addition to the structured search.

Stage 3: Study selection

The study selection process was conducted using Covidence software (Covidence systematic review software, Veritas Health Innovation, Melbourne, Australia). All papers identified in the literature search (n=771) were exported to the Covidence systematic review software, where duplications were sought and removed. Thereafter, all records were screened by the first and second authors (JB and KH) by reading the title and abstract. For inclusion, an original research paper had to report on

For peer review only

4

dynamic ambulance allocation for road ambulances and be published between 2012 and 2023. Both qualitative and quantitative studies were included. Papers reporting on air ambulance services, fixed locations of ambulance resources (such as the optimal location of an ambulance station), review studies, case studies or editorial texts were excluded. The full-text review assessed the entire paper and its eligibility for the study. In this stage of the selection process, the reason for exclusion was documented (see Figure 1). One disagreement occurred during the selection process, and it was solved by involving the second author (LK) in the decision.

In addition to the original scoping review methodology, a quality appraisal was performed [13], using the Critical Appraisal Skills Programme (CASPS) checklist for cohort studies [14]. The quality appraisal was performed by the first author (JB) and validated by the fourth author (KH) (see Supplementary Material 2).

Stage 4: Charting the data

All included studies were reviewed, and the following information was extracted: author(s) and year of publication, study location, design, aim, focus/ aim of the study, setting (urban and/or rural areas), type of modelling, variables used, main findings and quality appraisal.

Stage 5: Collating, summarising and reporting the results.

The results were synthesised and reported according to three domains; (1) study contexts and designs, (2) outcome variables and variables used in predictions of optimal ambulance dispatching and/ or relocation and (3) outcomes from dynamic ambulance relocation.

Patient and Public Involvement statement

No patients were included in the study.

Figure 1: Overview of the selection process and reasons for exclusion.

RESULTS

In total, 21 original papers were included in this scoping review. The included articles were published between 2012 and 2022, and the studies were conducted in Austria (n=1), Brazil (n=1), Canada (n=1), China (n=1), Colombia (n=1), Germany (n=1), Indonesia (n=1), Iran (n=2), Portugal (n=1), Singapore (n=3), Slovakia (n=1), Spain (n=1), Switzerland (n=1), the Netherlands (n=3), Turkey (n=1) and the USA (n=1). The studies were assessed as medium (n=16) or high (n=5) in quality (see Supplementary Material 2).

Study contexts and designs

The studies were most often conducted in urban areas, including metropolitan cities. Few studies were conducted in rural areas (see Supplementary Material 3). One study [15] was only based on emergency calls for critical patients.

All studies were based on mathematical simulation models, with a large variation of outcome variables. Most studies (n=12) used an experimental design and simulations. Of these, most (n=8) simulations were based on retrospective EMS data and medical emergencies within a specified region (see Table 1 and Supplementary Material 3). Seven studies used experimental design (theoretical applications with no simulation) and two studies reported a prospective, real-time

For peer review only

evaluation of the models and technical systems (see Supplementary Material 3). Two studies evaluated their developed models in real-time evaluations, one in both urban and rural areas [16] and one in an urban context [17].

Outcome variables and variables used in predictions of optimal ambulance dispatching and/ or relocation

Most studies (n=17) used response time as the primary outcome variable for the models developed and/ or tested. The information used to develop the mathematical models and/or computer-based programs was collected from retrospective data from real EMS activities (n=6), geographic and demographic data from national databases or Google Earth (n=6), estimations made by the research teams (n=3) or a combination of these sources within the same study (n=3) (see Supplementary Material 3). Some studies did not specify the sources for their variable data (n=10). All studies included three or more variables in their models.

Table 1. Summary of included studies

		Number of studies* N= 21
**Study design**	Experimental design and simulations	12
	Experimental design with no simulation/ testing	7
	Experimental design with real-time evaluation of models	2
**Data used**	Retrospective data from EMS activities	4
	Geographic and demographic data from national databases or Google Earth	8
	Estimations made by the research teams	3
	Combinations of estimations and official data	3
	No information given	3
**Study context**	Urban areas	12
	Rural areas	2
	Both urban and rural areas	4
	No information given	2
**Outcome variable**	Response time	17
	Turnaround time	3
	Workload on each ambulance	2
	Availability for new calls	2
	Perceived feasibility of software	1
	Travel distance per ambulance	1
**Variables used in the models**	Travel times and road network	14
	Current location of ambulances	13
	Number of available ambulances	9
	Location of hospitals	6
	Population living in a defined geographical area	6
	Time of day	4
	Response time	7
	Expected occurrence of medical emergencies within a specified area or timeframe	5
	Type of emergency or priority of the call	3
	Turnaround time	2
	Time at scene	2
	Uncovered emergencies	1
	Economical costs	1

For peer review only

6

	EMS working hours	4
	EMS workload	2
	Chain relocation effects	1

*Each study could include several variables

Outcomes from dynamic ambulance relocation

No study reported or discussed medical outcomes or clinical effects of dynamic relocation of ambulances. Four studies reported decreased response times when using dynamic relocation compared to ordinary dispatching and when using ordinary ambulance stations. Reported time saved was 130-160 seconds [18], 44 seconds [19], 54 seconds [20] and undefined saving of time [71]. In one study, the average percentage of calls reached within 9 min was improved with 43% up to 55% in different simulations [18]. However, the same study showed that an increase in total travelled distance by 7.9% to 31.7% [18].

No study reported on changes in response time or turnaround time in rural areas. Chain effects on other emergency calls due to dynamic ambulance relocations were reported [16]. One study reported that dynamic ambulance relocation reduced the total working time of all ambulances by about 9% per shift [17].

DISCUSSION

This review showed that most studies on dynamic ambulance relocation were based on experimental study designs and presented mathematical models in which three or more variables were used to calculate dynamic relocation of an ambulance with respect to response time. Most of the variables used in the models were fixed, and few studies evaluated the models or programs in prospective, real-life settings. Most studies were conducted in rural areas. No study reported on patient outcomes attained using dynamic ambulance relocation.

Ambulance dispatching and the optimal location of ambulances are important but challenging questions to analyse. Most variables used in the models presented were static, gathered from GIS systems or similar techniques. New techniques, such as artificial intelligence (AI), may offer additional tools for decision-making and dispatching of ambulances in the future [21]. However, dispatchers also use other variables, such as medical priority, type of medical care required, clinical judgement and their own professional experience, when making decisions on ambulance dispatching [22]. Therefore, an important consideration is to identify how fixed variables can be incorporated into decision-making systems, while also recognising the limitations of these tools.

No consensus has yet been reached on how to assess, define or measure the quality of care within the prehospital setting [23]; however, prehospital response times have long been a core component in these discussions [10]. Moreover, response times are often available for research and are (therefore) used as a proxy for outcome, which is also reflected in the results as most of the studies included in this review used response time as the primary outcome. However, trauma has traditionally been used as a role model for time-critical medical conditions, as prolonged response time is associated with increased mortality in trauma victims [24], especially in rural areas [11] . Studies suggest that prolonged prehospital time may have positive, negative and neutral associations with trauma patient mortality [25]. Patients with critical conditions, such as cardiac arrests, are likely to benefit from also small improvements in response time. However, the actual time gained by using dynamic ambulance relocations, in comparison to static ambulance allocation (e.g. the ordinary ambulance station), was only studied in three of the articles, and in those, the time saved rendered from a few seconds up to two minutes. Hence, whether this saved time had clinical relevance (i.e.

affected the outcome of the patients) cannot be determined as it was not measured. Dynamic ambulance relocation strategies focusing solely on shortening the average response time might be counter-productive when trying to save lives and minimize harm, especially in rural areas, since it may case longer response times or turnaround times for other patients. The goal must be to reach as many patients as possible with time-critical conditions "fast enough", not shortening the average response- or turnaround times. Reaching some patients fast enough might mean that other patients must wait longer for an ambulance. Less populated areas might have to rely on other rapid response units for the initial and potentially life-saving response before an ambulance can arrive. Since prehospital time is most likely not the only variable effecting clinical outcomes and quality of service within the ambulance services, variables such as early identification of time critical conditions, prehospital medical interventions provided [24] and the actual way of driving the ambulance [26] needs to be taken into consideration when discussing the *optimal* ambulance service. Studies evaluating the effect of ambulance relocation with respect to patient outcome with respect to the global effect of the relocation, ie not only on singular medical conditions but rather the effect on the patient population serviced by the relocated ambulances in real life contexts are strongly needed.

This review also does not allow any judgement of the value of dynamic relocation based on turnaround times, as only two of the included studies reported turnaround times. Both of these studies were conducted in large cities with many available emergency departments and neither compared turnaround times when dispatching using fixed ambulance locations versus dynamic relocation. Dynamic ambulance relocations may also cause a chain reaction, as temporary relocation of one ambulance may lead to a lack of resources in the original location, thus leading to new relocations and so on. These effects were covered in only one of the included studies. Therefore, no conclusions can be drawn regarding reductions in the response- or turnaround time by dynamic ambulance relocation based on the published studies.

Most studies developed and evaluated models based on EMS in urban areas. Notable differences exist between urban and rural areas regarding ambulance coverage, and turnaround time is most likely shorter in a city than in rural areas. A shortage of ambulance resources may therefore be more critical in rural areas than in urban areas. If the number of ambulances is small, the effect of a shortage or temporary relocation of one ambulance is more noticeable than in a city with more ambulances per area [27]. Conversely, the statistical risk for the occurrence of a time-critical condition is considerably lower in rural areas with small populations than in cities with large populations. However, the distances might be longer in rural areas than in urban areas. Even a temporary relocation may cause a significant delay in EMS response time; therefore, the effects of dynamic relocation might be more prominent in rural areas. Given the vulnerability of EMS coverage in rural areas, dynamic ambulance relocation in these areas needs to be prioritised in future studies. An important point made by Bélanger et al [18] is that dynamic ambulance relocation strategies could not compensate for a general lack of resources in a longer perspective and had side effects such as prolonged travelled distance and related costs. This was also the only identified study reflecting on optimal ambulance use from an economical or environmental perspective.

The well-being and working environment of EMS personnel is another relevant aspect of the optimal use of available ambulances. Long working hours, shift work and short times between the missions used for recovery are associated with work-related stress and mental health problems [28]. A practical consequence of dynamic ambulance relocation can be limited access to a toilet, kitchen or resting facilities for ambulance personnel. Hence, human resource management may be more challenging when using dynamic dispatch strategies compared to fixed ambulance locations [18]. In this review, only one model included variables associated with the ambulance personnel working

For peer review only

8

times, shifts and night rest. Only one study reported on workload effects. Working hours, working environment and workload are all important for both the effectiveness and efficiency of the EMS and should be considered when creating models for dynamic ambulance relocations.

Limitations

This scoping review has several limitations. One was that the search was performed in three databases. However, these provided a broad selection of both medical and non-medical journals and were therefore considered sufficient. By including studies from medical, technical and mathematical disciplines, the format for paper presentations varied. According to the scoping review methodology, only data from the results section should be charted and used in the review results. However, since the structure of the included papers varied, valuable information or outcomes stated under other headings were also extracted and included in the results of this review if the data or outcome referred to the actual study. The search strategy was revised several times during the revision process of this paper. The inconsistent terminology and definitions used for ambulance dispatching and dynamic relocation make it difficult to identify all available papers and motivated a free text search in addition to the systematic searches. A quality appraisal is not mandatory when using the scoping review methodology. However, to judge the value of the findings, a quality appraisal was found to add value and was therefore conducted.

CONCLUSION

The current knowledge on dynamic relocation emanates from mathematical and technical support used to calculate the optimal locations of ambulance services. However, knowledge of how patient outcomes and working environments are affected by dynamic ambulance dispatching is lacking. This review has highlighted several gaps in the scientific coverage of the topic. The primary concerns are the lack of studies reporting on patient outcomes and the limited knowledge about several key factors, including the optimal use of ambulances in rural areas, turnaround times, chain effects and aspects of working environment for EMS personnel. Therefore, addressing these knowledge gaps will be important in future studies.

List of abbreviations

Emergency Medical Services (EMS)

PRISMA Extension for Scoping Reviews (PRISMA-ScR)

Funding

This research received no specific grant from any funding agency in the public, commercial or not-for-profit sectors.

Acknowledgements

The authors would like to thank academic librarians Ulrika Johansson (Örebro University library) and Lina Puhakka (Örebro University library) for their support in the literature search process.

DECLARATIONS

Ethics approval and consent to participate

This study does not involve participants; therefore, ethical approval or consent was not required.

Availability of data and materials

For peer review only

9

Data sharing was not applicable, as no data sets were generated and/or analysed in this study.

Competing interests
The authors declare that they have no competing interests.

Author contributions
JB: Conceptualization, data collection, analysis, writing of the manuscript LK: Conceptualization, analysis, writing of the manuscript, EH: Conceptualization, data collection, confirming analysis, writing of the manuscript, KH: Conceptualization, data collection, analysis, writing and editing of the manuscript and revision. All authors read and approved the final manuscript.

References

1. Andrew E, Nehme Z, Cameron P, *et al.* Drivers of increasing emergency ambulance demand. *Prehosp Emerg Care* 2020;24(3):385-93. doi: 10.1080/10903127.2019.1635670 [published Online First: 2019/06/27]
2. Bélanger V, Ruiz A, Soriano P. Recent optimization models and trends in location, relocation, and dispatching of emergency medical vehicles. *Eur J Oper Res* 2019;272(1):1-23. doi: 10.1016/j.ejor.2018.02.055
3. Jánošíková L, Jankovič P, Kvet M, *et al.* Reorganization of an emergency medical system in a mixed urban-rural area. *Int J Environ Res Public Health* 2022;19(19):12369. doi: 10.3390/ijerph191912369 [published Online First: 2022/10/15]
4. Schmid V, Doerner KF. Ambulance location and relocation problems with time-dependent travel times. *Eur J Oper Res* 2010;207(3):1293-303. doi: 10.1016/j.ejor.2010.06.033
5. Nysam. Ambulanssjukvård. Nyckeltal 2019. Rapport Maj 2020. (In English: Ambulance care. Statistics 2019. Report May 2020). Stockholm, 2020. Available at: https://nysam.com/images/Rapporter/Ambulanssjukvard_2019_Nysamrapport.pdf
6. Örebro County Council AD. Statistics. Örebro, Sweden, 2023.
7. Neira-Rodado D, Escobar-Velasquez JW, McClean S. Ambulances deployment problems: categorization, evolution and dynamic problems review. *ISPRS Int J Geo-Inf* 2022;11(2):109. doi: 10.3390/ijgi11020109
8. Chen X, Guyette FX, Peitzman AB, *et al.* Identifying patients with time-sensitive injuries: Association of mortality with increasing prehospital time. *J Trauma Acute Care Surg* 2019;86(6):1015-22. doi: 10.1097/ta.0000000000002251 [published Online First: 2019/05/28]
9. van Rein EAJ, van der Sluijs R, Houwert RM, *et al.* Effectiveness of prehospital trauma triage systems in selecting severely injured patients: Is comparative analysis possible? *Am J Emerg Med* 2018;36(6):1060-69. doi: 10.1016/j.ajem.2018.01.055 [published Online First: 2018/02/06]
10. El Sayed MJ. Measuring quality in emergency medical services: a review of clinical performance indicators. *Emerg Med Int* 2012;2012:161630. doi: 10.1155/2012/161630 [published Online First: 2011/11/03]
11. Harmsen AM, Giannakopoulos GF, Moerbeek PR, *et al.* The influence of prehospital time on trauma patients outcome: a systematic review. *Injury* 2015;46(4):602-9. doi: 10.1016/j.injury.2015.01.008 [published Online First: 2015/01/30]
12. Arksey H, O'Malley, L. Scoping studies: towards a methodological framework. *Int J Soc Res Methodol: Theory & Pract* 2005;8(1):19-32. doi: 10.1080/1364557032000119616
13. Sucharew H, Macaluso M. Progress notes: methods for research evidence synthesis: the scoping review approach. *J Hosp Med* 2019;14(7):416-18. doi: 10.12788/jhm.3248 [published Online First: 2019/06/30]

For peer review only

10

14. (CASP) CASP. CASP Coort Study Checklists Available at: https://casp-uk.net/casp-tools-checklists/: CASP; 2020 [accessed November 04 2020].
15. Billhardt H, Lujak M, Sánchez-Brunete V, *et al.* Dynamic coordination of ambulances for emergency medical assistance services. *Knowl Based Syst* 2014;70:268-80. doi: 10.1016/j.knosys.2014.07.006
16. van Buuren M, Jagtenberg, C., van Barneveld, T.,van der Mei, R., Bhulai, S. Ambulance dispatch center pilots proactive relocation policies to enhance effectiveness. *Interfaces* 2018;48(3):235-46. doi: 10.1287/inte.2017.0936
17. Hajiali M, Teimoury E, Rabiee M, *et al.* An interactive decision support system for real-time ambulance relocation with priority guidelines. *Decis Support Syst* 2022;155:113712. doi: 10.1016/j.dss.2021.113712
18. Bélanger V, Kergosien Y, Ruiz A, Soriano P. An empirical comparison of relocation strategies in real-time ambulance fleet management. *Comput. Ind. Eng*. 2016;94:216-29. doi.org:/10.1016/j.cie.2016.01.023
19. Lam SS, Zhang J, Zhang ZC, *et al.* Dynamic ambulance reallocation for the reduction of ambulance response times using system status management. *Am J Emerg Med* 2015;33(2):159-66. doi: 10.1016/j.ajem.2014.10.044 [published Online First: 2014/12/10]
20. Deng Y, Zhang Y, Pan J. Optimization for locating emergency medical service facilities: a case study for health planning from China. *Risk Manag Healthc Policy* 2021;14:1791-802. doi: 10.2147/rmhp.S304475 [published Online First: 2021/05/11]
21. Ceklic E, Ball S, Finn J, *et al.* Ambulance dispatch prioritisation for traffic crashes using machine learning: A natural language approach. *Int J Med Inform* 2022;168:104886. doi: 10.1016/j.ijmedinf.2022.104886 [published Online First: 2022/10/29]
22. Hosseini SMR, Maleki M, Gorji HA, *et al.* Factors affecting emergency medical dispatchers' decision-making: a qualitative study. *J Multidiscip Healthc* 2018;11:391-98. doi: 10.2147/jmdh.S159593 [published Online First: 2018/09/04]
23. Rehn M, Kruger AJ. Quality improvement in pre-hospital critical care: increased value through research and publication. *Scand J Trauma, Resusc Emerg Med* 2014;22:34. doi: 10.1186/1757-7241-22-34 [published Online First: 2014/06/03]
24. Brown JB, Rosengart MR, Forsythe RM, *et al*. Not all prehospital time is equal: Influence of scene time on mortality. *J Trauma Acute Care Surg* 2016;81(1):93-100. doi: 10.1097/ta.0000000000000999 [published Online First: 2016/02/18]
25. Bedard AF, Mata LV, Dymond C, *et al*. A scoping review of worldwide studies evaluating the effects of prehospital time on trauma outcomes. *Int J Emerg Med* 2020;13(1):64. doi: 10.1186/s12245-020-00324-7 [published Online First: 2020/12/11]
26. Becker J, Hugelius K. Driving the ambulance: an essential component of emergency medical services: an integrative review. *BMC emerg med*. 2021;21(1):160. doi: 10.1186/s12873-021-00554-9.
27. van Barneveld TC, Bhulai S, van der Mei RD. A dynamic ambulance management model for rural areas : Computing redeployment actions for relevant performance measures. *Health Care Manag Sci* 2017;20(2):165-86. doi: 10.1007/s10729-015-9341-3 [published Online First: 2015/10/05]
28. Lawn S, Roberts L, Willis E, *et al.* The effects of emergency medical service work on the psychological, physical, and social well-being of ambulance personnel: a systematic review of qualitative research. *BMC Psychiatry* 2020;20(1):348. doi: 10.1186/s12888-020-02752-4 [published Online First: 2020/07/06]

For peer review only

11

For peer review only

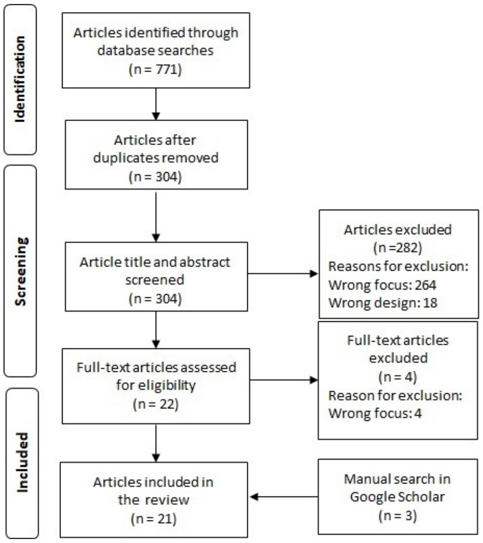

Figure 1. Overview of inclusion process

169x90mm (144 x 144 DPI)

Supplementary material 1; Search strategy

Search strategy for each database

Pubmed (NLM). Search performed October 12, 2023, 338 papers.

Ambulance, emergency medical services, dispatch, dynamic, dynamic allocation, relocation, machine learning		
	1.	Search: ((((dispatch*[Title/Abstract]) OR ("Emergency Medical Dispatcher"[Mesh])) OR ("Resource Allocation"[Mesh]) OR ("Emergency Medical Dispatch"[Mesh]) OR (allocation[Title/Abstract])) OR (relocation[Title/Abstract])) OR (reallocation[Title/Abstract]) OR (redeployment[Title/Abstract]) OR (deployment[Title/Abstract]) AND ((((((((((algorithm*[Title/Abstract]) OR (artificial intelligence[Title/Abstract])) OR (AI[Title/Abstract])) OR (machine learning[Title/Abstract])) OR (data-driven[Title/Abstract])) OR (data-based[Title/Abstract])) OR ("dynamic allocation"[Title/Abstract]) OR ("dynamic*"[Title/Abstract])) OR ("Algorithms"[Mesh])) OR ("Artificial Intelligence"[Mesh])) OR ("Machine Learning"[Mesh]))) AND ((((((ambulance*[Title/Abstract]) OR ("emergency medical service*"[Title/Abstract])) OR ("emergency service*"[Title/Abstract])) OR (prehospital[Title/Abstract])) OR ("Ambulances"[Mesh])) OR ("Emergency Medical Services"[Mesh]))
	2.	1 AND Filters: English, from 2012 - 2023

Web of Science Core Collection (Clarivate) (1975-present) Search performed October 19, 2023, 301 papers.

Indexes=SCI-EXPANDED, SSCI, A&HCI, CPCI-S, CPCI-SSH, ESCI Timespan=All years

Ambulance, emergency medical service		
	1.	ALL=(((ambulance OR emergency medical service)))
Dispatch		
	2.	ALL=((dispatch*OR allocation* OR reallocation* OR redeployment* OR deployment*))
Dynamic, relocation, machine learning		
	3.	ALL=(((dynamic* OR dynamic allocation OR relocation OR machine learning OR data-driven OR artificial intelligence OR Machine learning)))
Combined Sets		
	4.	1 AND 2 AND 3
Limits		
	6.	4 and English (Languages) and 2023 or 2022 or 2021 or 2020 or 2019 or 2018 or 2017 or 2016 or 2015 or 2014 or 2013 or 2012 (Publication Years)

For peer review only

Scopus (Elsevier). Search performed October 19, 132 abstracts

Ambulance, emergency medical services		
	1.	(TITLE-ABS-KEY (ambulance*) OR TITLE-ABS-KEY (emergency AND medical AND service*))
Dispatch		
	2.	TITLE-ABS-KEY (dispatch*)
Dynamic, relocation, machine learning		
	3.	(TITLE-ABS-KEY (dynamic*) OR TITLE-ABS-KEY (relocation*) OR TITLE-ABS-KEY (machine AND learning)) OR TITLE-ABS-KEY (dynamic AND allocation) OR TITLE-ABS-KEY (relocation) OR TITLE-ABS-KEY (machine learning) OR TITLE-ABS-KEY (data-driven) OR TITLE-ABS-KEY (artificial intelligence)))
Combined Sets		
	4.	1 AND 2 AND 3
Limits		
	8.	4 AND (LIMIT-TO (LANGUAGE , "English")AND PUBYEAR > 2011 AND PUBYEAR < 2024)

Google Scholar Search performed September 12 and October 20, 2023. Manual search.

Dynamic ambulance		
	1.	'dynamic ambulance'
Ambulance relocation		
	2.	'ambulance relocation'
	3.	'dynamic ambulance dispatch'
Limits		
	4.	(Publication Years 2012-2023). Sorted: Best match.

For peer review only

Supplementary material 2: Quality appraisal according to CASP protocols

Author/ year	Did the study address a clearly focused issue?	Was the cohort requited in an acceptable way?	Was the exposure accurately measured to minimize bias?	Was the outcome accurately measured to minimize bias?	Have the authors identified all important confounding factors?	Have con-founding factors in the design and/or analysis been taken into consideration?	Was the follow-up of subjects complete and long enough?	How precise are the results?	Do you believe in the results?	Can the results be applied to the local population?	Do the results of this study fit with other available evidence?	Overall quality
Barneveldt 2017	Yes	N/A	N/A	Some	Some	Some	Not able to judge	Some	Some	Yes	Yes	Medium
Bélanger et al 2016	Yes	N/A	N/A	Yes	Some	Yes	Not able to judge	Some	Yes	Yes	Yes	Medium
Billhart et al 2014	Yes	N/A	N/A	Some	No	Some	Yes	Some	Yes	Yes	Yes	Medium
Carvallo et al 2020	Yes	N/A	N/A	Some	Some	Yes	Yes	Some	Yes	Yes	Yes	High
Degel et al 2015	Yes	N/A	N/A	Some	Some	Yes	Yes	Some	Yes	Yes	Yes	Medium
Deng et al 2021	Yes	N/A	N/A	Some	Some	Some	N/A	Some	Some	Unclear	Yes	Medium
Enayati et al 2018	Yes	N/A	N/A	Some	Some	Some	Yes	Some	Some	Yes	Yes	Medium
Hajiali et al 2022	Yes	N/A	N/A	Yes	Some	Yes	No	Good	Some	Yes	Some	High
Jagtenberg et al 2017	Yes	N/A	N/A	Some	Some	Yes	N/A	Some	Some	Unclear	Yes	Medium
Jánošíková et al 2021	Yes	N/A	N/A	Some	Some	Some	Not able to judge	Some	Some	Yes	Yes	Medium
Karpova et al 2022	Yes	N/A	N/A	Some	Some	Some	Not able to judge	Some	Some	Yes	Yes	Medium
Lam et al 2014	Yes	N/A	N/A	Yes	Yes	Some	Yes	Good	Yes	Yes	Yes	High
Lam et al 2015	Yes	N/A	N/A	Yes	Some	Some	Yes	Some	Yes	Yes	Yes	Medium
Lam et al 2017	Yes	N/A	N/A	Yes	Yes	Yes	Yes	Some	Yes	Yes	Yes	High
Nogueira et al 2016	Yes	N/A	N/A	Some	Some	Some	Some	Some	Some	Yes	Yes	Medium
Roa et al 2020	Yes	N/A	N/A	Some	Some	Some	Some	Some	Some	Yes	Yes	Medium
Schmid 2021	Yes	N/A	N/A	Some	Some	Some	Yes	Some	Some	Yes	Yes	Medium
Strauss et al 2021	Yes	N/A	N/A	Some	Some	Some	Unclear	Some	Yes	Yes	Yes	Medium

For peer review only

Swalehe & Aktas 2016	Yes	N/A	N/A	Some	Some	Some	Unclear	Some	Yes	Yes	Yes	Medium
Umam et al 2022	Yes	N/A	N/A	Some	Some	Some	Unclear	Some	Yes	Yes	Yes	Medium
Van Buuren et al 2018	Yes	N/A	N/A	Yes	Some	Yes	Yes	Good	Yes	Yes	Yes	High

For peer review only

Supplementary material 3. Overview of included studies

Authors and publication year	Study location	Design	Aim of the study	Setting	Type of modelling	Variables used in the model	Outcome variable	Main findings	Quality appraisal
Barneveldt et al 2017[1]	The Netherlands	Experimental	To develop a method to obtain good relocation strategies in real time	Rural areas	Mathematic models and scenario probabilities based on retrospective data	Demand locations<br>Time of the day<br>Level of priority<br>Total number of ambulances<br>Treatment time on scene	Response time	A one-step look-ahead heuristic model that relocated ambulances to minimize the response time was presented	Medium quality
Bélanger et al 2016[2]	Canada	Experimental with simulation	To study outcomes of different location and relocation strategies.	Urban areas	Mathematic models and scenario probabilities	Available ambulances<br>Travel times<br>Ambulance stations locations<br>Population density<br>Expected emergency calls<br>Working shift time	Response time<br>Response rate within 9 minutes<br>Travelled distance	Using a flexible relocation strategy provides a better service. However, when the system is congested, dynamic strategies cannot compensate for the lack of resources	Medium quality
Billhardt et al 2014[3]	Spain	Experimental	To present an event-driven system for dynamic ambulance assignment and redeployment	Urban areas	Mathematic models and scenario probabilities	Geographical location<br>Available ambulances<br>(The study only included critical patients)	Response time	Mathematic models can reduce the response times for EMS services. However, more variables need to be considered and included in such models	Medium quality

For peer review only

Carvalho et al 2020[4]	Portugal	Experimental with simulation	To develop models to solve ambulance dispatching and relocation problems	Urban areas	Mathematic models and scenario probabilities based on retrospective data (n=20 000)	Available ambulances<br>Travel times<br>Ambulance stations locations<br>Dynamic relocation spots<br>Waiting time<br>Maximum response times<br>Uncovered emergencies	Availability for new calls	A mixed-integrated programming model and an experimental model was presented. Both approaches used a time preparedness measure to evaluate the system's capability to handle new emergencies	High quality
Degel et al 2015[5]	Germany	Experimental with simulation	To support decision on location planning for ambulances	Rural areas	Mathematic models and scenario probabilities based on retrospective data	Travel time<br>Required coverage<br>Variation in fleet<br>Flexible locations	Availability for new calls	The inclusion of time-dependent and empirical data had a substantial impact on the solution	Medium quality
Deng et al 2021[6]	China	Experimental	To optimize the EMS system	No information given	Mathematic models	Population density<br>Road network<br>Location of hospitals<br>Location of flexible locations	Response time	The solution presented could effectively achieve a population coverage within 15 minutes, with a minimum number of temporary ambulance points in addition to existing	Medium quality

For peer review only

								ambulance stations	
Enayati et al 2018[7]	USA	Experimental with simulation	To develop a real-time model to maximize coverage with minimum possible total travel time	Urban	Mathematic models and scenario probabilities based on retrospective data (n=50 000)	Initial fleet size of ambulances<br>Travel limit (max 20 min)<br>Maximum allowed workload of each ambulance<br>Day and time<br>Shift times (12h)	Turnaround time	The modeling of the real time deployment model resulted in improvement in average coverage and prevented accumulated workload for ambulance personnel	Medium quality
Hajiali et al, 2022[8]	Iran	Experimental real-life testing	To develop a decision support system for optimal ambulance coverage	Urban	Mathematic models and real-life evaluation (1 week) of response time and workload for each ambulance	Base location<br>Demand zones and time ranges from these (7 min, 15 min, 25 min)<br>Number of available ambulances<br>Relocation time<br>Working shift time (12h)	Response time<br>Workload	The decision support system decreased response times and reduced the total working time of all ambulances by about 9% per shift.	Hight quality
Jagtenberg et al 2017[9]	The Netherlands	Experimental with simulation	To present different dispatch rules and suggest a heuristic method for ambulance dispatching	Urban areas	Mathematic models and scenario probabilities	Ambulance station locations<br>Demand locations<br>Hospital locations<br>the base location<br>Time of the day<br>Driving time	Response time	Two methods to obtain ambulance dispatch policies were reported. Their effects could not be defined	Medium quality
Jánošíková et al 2021[10]	Slovakia	Experimental with simulation	To identify optimization	Urban and rural areas	Mathematic models and	Demand zones<br>Population (and age profile)	Response time	The EMS systems should be designed to	Medium quality

For peer review only

			criterion of the EMS		scenario probabilities	Temporary locations Travel time		minimize response times, and not to maximize the number of calls served within a given time limit	
Karpova et al 2022 (2023)[11]	Spain	Experimental	To addresses the problem of dynamic relocation of ambulances through the design and development of heuristic tools	Urban and rural areas	Mathematic models and scenario probabilities	Population coverage in demand zones<br>Ordinary base location<br>Driving time<br>Possible relocation bases	Response time	The designed relocation algorithms perform better than if there was no relocation strategy.	Medium quality
Lam et al 2014[12]	Singapore	Experimental with simulation	To develop a discrete-event simulation model for the Singapore EMS	Urban area	Mathematic models and scenario probabilities based on retrospective data (n=54 548)	Geographical information systems (GIS) data, Travel time<br>Ambulance location	Response time	Response times were improved via a more effective reallocation of ambulances and dispatch policy, without additional ambulances	High quality
Lam et al 2015[13]	Singapore	Experimental with simulation	To develop efficient ambulance allocation plans in Singapore and to evaluate its	Urban area	Mathematic models and scenario probabilities based on retrospective data (n= 52 512)	Call times<br>Dispatch times,<br>Scene arrival times,<br>Hospital conveyance times<br>Turnaround time,<br>Locations of incident	Response time	Geographical information system-based plans reduced the median response times with 13 to 44 seconds	Medium

For peer review only

			performance using the DES model.					compared to a static reallocation policy	
Lam et al 2017[14]	Singapore	Experimental with simulation	To report on the previously reported work by leveraging on the DES model	Urban area	Mathematic models and scenario probabilities based on retrospective data (n=217 000)	Geographical coverage<br>Response Times<br>Turnaround times and utilization rates	Turnaround time	The integration of dynamic redeployments optimized the ambulance coverage	High quality
Nogueira et al 2016[15]	Brazil	Experimental with simulation	To analyze the EMS using two modeling techniques: optimization and simulation	Urban areas	Mathematic models and scenario probabilities	Ambulance station location<br>Demand posts<br>Hospital locations<br>Cost for each ambulance	Response time	A balanced number of ambulances allocated across certain bases at certain times improved the system performance without increasing the total number of ambulances	Medium quality
Roa et al 2020[16]	Colombia	Experimental with simulation	To design an algorithm to support the real-time operation of ambulances	Urban areas	Mathematic models and scenario probabilities based on retrospective data	Types of emergency,<br>Types of ambulances<br>Demand points<br>Locations for ambulances<br>Modelled on-site attention time<br>Travel times	Response time<br>Availability for new calls	The real-time management of ambulance fleets involved challenging decisions due to frequent, complex, and random changes in system conditions	Medium quality

Schmid 2021[17]	Austria	Experimental with simulation	To propose a stochastic dynamic model for the ambulance relocation and dispatching problem	Urban area	Mathematic models and scenario probabilities based on retrospective data (n=3748)	Location of hospitals and patients, response times, travel times, using data.	Response time	By deviating from the traditional rule of dispatching the closest ambulance available and relocating them to their home base after having finished serving, the coverage can be improved	Medium quality
Strauss et al 2021[18]	Switzerland and Germany	Experimental with simulation	To explain the major features of "rule-based discrete event simulation"	Urban and rural areas	Mathematic models and scenario probabilities	Response times, demands per day, travel time, dispatch priority	Response time	Rule-based DES models can improve regional emergency services' efficiency without increasing cost. All simulation-based methods suggest normative solutions and optimize EMS' performance within given healthcare system structures	Medium quality
Swalehe & Aktas 2016[19]	Turkey	Experimental	To find optimal ambulance	Urban	Computer based systems (one with	Time of day Population Driving time	Response time	Ambulance response time can be reduced	Medium quality

			locations (stations and temporary demand points)		information on retrospective emergency calls	Road network data		with a dynamic relocation plan taking time of the day in consideration.<br>Dynamic ambulance deployment is a more effective ambulance deployment strategy than static ambulance deployment	
Umam et al 2022[20]	Indonesia	Experimental	To overcome the location problem and allocation of ambulances by using a combination of metaheuristics and simulation	No information given	Mathematic models	Ambulance station location<br>Demand posts<br>Travel times<br>Number of available ambulances	Response time	Involving several potential bases can produce a short response time.	Medium quality
Van Buuren et al 2018[21]	The Netherlands	Experimental real-life testing	To evaluate two dynamic relocation policies for operational use and implemented in a software tool for realtime	Urban and rural areas	Mathematic models and real-life evaluation (qual and quant)	Geographical distances<br>Population<br>Probability of new medical emergencies<br>Ambulance stations<br>Travel times<br>Chain relocation effects EMS sleepingtimes	Response time<br>Feasibility of the software	When dispatchers worked in a consistent way, relocation decisions were made faster, dispatchers had a better overview of the	High quality

For peer review only

	decision support		EMS shiftworking times	available ambulances. The use of policies instead of dispatcher intuition improved efficiency.

References for included studies

1. van Barneveld TC, Bhulai S, van der Mei RD. A dynamic ambulance management model for rural areas : Computing redeployment actions for relevant performance measures. Health Care Manag Sci 2017;20(2):165-86. doi: 10.1007/s10729-015-9341-3 [published Online First: 2015/10/05]

2. Bélanger V, Kergosien Y, Ruiz A, Soriano P. An empirical comparison of relocation strategies in real-time ambulance fleet management. Comput Ind Eng. 2016;94:216-29.

3. Billhardt H, Lujak M, Sánchez-Brunete V, et al. Dynamic coordination of ambulances for emergency medical assistance services. Knowledge-Based Systems 2014;70:268-80. doi: https://doi.org/10.1016/j.knosys.2014.07.006

4. Carvalho AS, Captivo ME, Marques I. Integrating the ambulance dispatching and relocation problems to maximize system's preparedness. European Journal of Operational Research 2020;283(3):1064-80. doi: https://doi.org/10.1016/j.ejor.2019.11.056

5. Degel D, Wiesche L, Rachuba S, et al. Time-dependent ambulance allocation considering data-driven empirically required coverage. Health Care Manag Sci 2015;18(4):444-58. doi: 10.1007/s10729-014-9271-5 [published Online First: 2014/03/13]

6. Deng Y, Zhang Y, Pan J. Optimization for Locating Emergency Medical Service Facilities: A Case Study for Health Planning from China. Risk Manag Healthc Policy 2021;14:1791-802. doi: 10.2147/rmhp.S304475 [published Online First: 2021/05/11]

7. Enayati S, Mayorga ME, Rajagopalan HK, et al. Real-time ambulance redeployment approach to improve service coverage with fair and restricted workload for EMS providers. Omega 2018;79:67-80. doi: https://doi.org/10.1016/j.omega.2017.08.001

8. Hajiali M, Teimoury E, Rabiee M, et al. An interactive decision support system for real-time ambulance relocation with priority guidelines. Decision Support Systems 2022;155:113712. doi: https://doi.org/10.1016/j.dss.2021.113712

9. Jagtenberg CJ, Bhulai S, van der Mei RD. Dynamic ambulance dispatching: is the closest-idle policy always optimal? Health Care Manag Sci 2017;20(4):517-31. doi: 10.1007/s10729-016-9368-0 [published Online First: 2016/05/22]

For peer review only

10. Jánošíková Ľ, Jankovič P, Kvet M, et al. Coverage versus response time objectives in ambulance location. Int J Health Geogr 2021;20(1):32. doi: 10.1186/s12942-021-00285-x [published Online First: 2021/07/04]

11. Karpova Y, Villa F, Vallada E, Vecina MÁ. Heuristic algorithms based on the isochron analysis for dynamic relocation of medical emergency vehicles. Expert Syst. Appl. 2023;212:118773. Available online Sept 2022.

12. Wei Lam SS, Zhang ZC, Oh HC, et al. Reducing ambulance response times using discrete event simulation. Prehospital emergency care : official journal of the National Association of EMS Physicians and the National Association of State EMS Directors 2014;18(2):207-16. doi: 10.3109/10903127.2013.836266 [published Online First: 2013/10/19]

13. Lam SS, Zhang J, Zhang ZC, et al. Dynamic ambulance reallocation for the reduction of ambulance response times using system status management. The American journal of emergency medicine 2015;33(2):159-66. doi: 10.1016/j.ajem.2014.10.044 [published Online First: 2014/12/10]

14. Lam SSW, Ng CBL, Nguyen F, et al. Simulation-based decision support framework for dynamic ambulance redeployment in Singapore. Int J Med Inform 2017;106:37-47. doi: 10.1016/j.ijmedinf.2017.06.005 [published Online First: 2017/09/06]

15. Nogueira LC, Jr., Pinto LR, Silva PM. Reducing Emergency Medical Service response time via the reallocation of ambulance bases. Health Care Manag Sci 2016;19(1):31-42. doi: 10.1007/s10729-014-9280-4 [published Online First: 2014/04/20]

16. Roa JC, Escobar, J.W., Marín- Moreno, C.A. An online real-time matheuristic algorithm for dispatch and relocation of ambulances. International Journal of Industrial Engineering Computations 2020;11(3):443-68.

17. Schmid V. Solving the dynamic ambulance relocation and dispatching problem using approximate dynamic programming. Eur J Oper Res 2012;219(3):611-21. doi: 10.1016/j.ejor.2011.10.043 [published Online First: 2012/06/16]

18. Strauss C, Bildstein G, Efe J, et al. Optimizing Emergency Medical Service Structures Using a Rule-Based Discrete Event Simulation-A Practitioner's Point of View. Int J Environ Res Public Health 2021;18(5) doi: 10.3390/ijerph18052649 [published Online First: 2021/04/04]

19. Swalehe M, Aktas SG. Dynamic Ambulance Deployment to Reduce Ambulance Response Times Using Geographic Information Systems: A Case Study of Odunpazari District of Eskisehir Province, Turkey. Procedia Environmental Sciences 2016;36:199-206. doi: https://doi.org/10.1016/j.proenv.2016.09.033

20. Umam, M. I. H., Santosa, B., & Siswanto, N. (2022). A Simulation Optimization for Location and Allocation of Emergency Medical Service. International Int. j. online biomed. eng, 18(11), pp. 158–172.

21. van Buuren M, Jagtenberg, C., van Barneveld, T.,van der Mei, R., Bhulai, S. Ambulance Dispatch Center Pilots Proactive Relocation Policies to Enhance Effectiveness. Interfaces 2018;48(3):235-46.

For peer review only

For peer review only

Preferred Reporting Items for Systematic reviews and Meta-Analyses extension for Scoping Reviews (PRISMA-ScR) Checklist

SECTION	ITEM	PRISMA-ScR CHECKLIST ITEM	REPORTED ON PAGE #
**TITLE**			
Title	1	Identify the report as a scoping review.	1
**ABSTRACT**			
Structured summary	2	Provide a structured summary that includes (as applicable): background, objectives, eligibility criteria, sources of evidence, charting methods, results, and conclusions that relate to the review questions and objectives.	2
**INTRODUCTION**			
Rationale	3	Describe the rationale for the review in the context of what is already known. Explain why the review questions/objectives lend themselves to a scoping review approach.	3
Objectives	4	Provide an explicit statement of the questions and objectives being addressed with reference to their key elements (e.g., population or participants, concepts, and context) or other relevant key elements used to conceptualize the review questions and/or objectives.	3
**METHODS**			
Protocol and registration	5	Indicate whether a review protocol exists; state if and where it can be accessed (e.g., a Web address); and if available, provide registration information, including the registration number.	N/A
Eligibility criteria	6	Specify characteristics of the sources of evidence used as eligibility criteria (e.g., years considered, language, and publication status), and provide a rationale.	4
Information sources*	7	Describe all information sources in the search (e.g., databases with dates of coverage and contact with authors to identify additional sources), as well as the date the most recent search was executed.	4
Search	8	Present the full electronic search strategy for at least 1 database, including any limits used, such that it could be repeated.	4
Selection of sources of evidence†	9	State the process for selecting sources of evidence (i.e., screening and eligibility) included in the scoping review.	4
Data charting process‡	10	Describe the methods of charting data from the included sources of evidence (e.g., calibrated forms or forms that have been tested by the team before their use, and whether data charting was done independently or in duplicate) and any processes for obtaining and confirming data from investigators.	4
Data items	11	List and define all variables for which data were sought and any assumptions and simplifications made.	4, Table 2
Critical appraisal of individual sources of evidence§	12	If done, provide a rationale for conducting a critical appraisal of included sources of evidence; describe the methods used and how this information was used in any data synthesis (if appropriate).	Table 2, Supple matrl
Synthesis of results	13	Describe the methods of handling and summarizing the data that were charted.	4

For peer review only - http://bmjopen.bmj.com/site/about/guidelines.xhtml

SECTION	ITEM	PRISMA-ScR CHECKLIST ITEM	REPORTED ON PAGE #
**RESULTS**			
Selection of sources of evidence	14	Give numbers of sources of evidence screened, assessed for eligibility, and included in the review, with reasons for exclusions at each stage, ideally using a flow diagram.	5
Characteristics of sources of evidence	15	For each source of evidence, present characteristics for which data were charted and provide the citations.	5-6
Critical appraisal within sources of evidence	16	If done, present data on critical appraisal of included sources of evidence (see item 12).	Supplementary
Results of individual sources of evidence	17	For each included source of evidence, present the relevant data that were charted that relate to the review questions and objectives.	Table 2
Synthesis of results	18	Summarize and/or present the charting results as they relate to the review questions and objectives.	5-6
**DISCUSSION**			
Summary of evidence	19	Summarize the main results (including an overview of concepts, themes, and types of evidence available), link to the review questions and objectives, and consider the relevance to key groups.	8
Limitations	20	Discuss the limitations of the scoping review process.	8
Conclusions	21	Provide a general interpretation of the results with respect to the review questions and objectives, as well as potential implications and/or next steps.	8
**FUNDING**			
Funding	22	Describe sources of funding for the included sources of evidence, as well as sources of funding for the scoping review. Describe the role of the funders of the scoping review.	8

JBI = Joanna Briggs Institute; PRISMA-ScR = Preferred Reporting Items for Systematic reviews and Meta-Analyses extension for Scoping Reviews.
* Where *sources of evidence* (see second footnote) are compiled from, such as bibliographic databases, social media platforms, and Web sites.
† A more inclusive/heterogeneous term used to account for the different types of evidence or data sources (e.g., quantitative and/or qualitative research, expert opinion, and policy documents) that may be eligible in a scoping review as opposed to only studies. This is not to be confused with *information sources* (see first footnote).
‡ The frameworks by Arksey and O'Malley (6) and Levac and colleagues (7) and the JBI guidance (4, 5) refer to the process of data extraction in a scoping review as data charting.
§ The process of systematically examining research evidence to assess its validity, results, and relevance before using it to inform a decision. This term is used for items 12 and 19 instead of "risk of bias" (which is more applicable to systematic reviews of interventions) to include and acknowledge the various sources of evidence that may be used in a scoping review (e.g., quantitative and/or qualitative research, expert opinion, and policy document).

From: Tricco AC, Lillie E, Zarin W, O'Brien KK, Colquhoun H, Levac D, et al. PRISMA Extension for Scoping Reviews (PRISMAScR): Checklist and Explanation. Ann Intern Med. 2018;169:467–473. doi: 10.7326/M18-0850.

For peer review only
